# Supplementary material for: Shadow-wall lithography of ballistic superconductor–semiconductor quantum devices
Source: Nat Commun. 2021 Aug 13;12:4914. doi: 10.1038/s41467-021-25100-w (PMC8363628; doi:10.1038/s41467-021-25100-w)
Supplement: Supplementary file 1 — Supplementary Information [file 41467_2021_25100_MOESM1_ESM.pdf]

Supplementary Information: Shadow-wall lithography of ballistic superconductor–semiconductor quantum devices

Sebastian Heedt*,[1,2,†] Marina Quintero-Pérez*,[2] Francesco Borsoi*,[1] Alexandra Fursina,[2] Nick van Loo,[1] Grzegorz P. Mazur,[1] Michał P. Nowak,[3] Mark Ammerlaan,[1] Kongyi Li,[1] Svetlana Korneychuk,[1] Jie Shen,[1] May An Y. van de Poll,[1] Ghada Badawy,[4] Sasa Gazibegovic,[4] Nick de Jong,[1,5] Pavel Aseev,[2] Kevin van Hoogdalem,[2] Erik P. A. M. Bakkers,[4] and Leo P. Kouwenhoven[1,2]

[1] *QuTech and Kavli Institute of Nanoscience, Delft University of Technology, 2600 GA Delft, The Netherlands*
[2] *Microsoft Quantum Lab Delft, 2600 GA Delft, The Netherlands*
[3] *AGH University of Science and Technology, Academic Centre for Materials and Nanotechnology, al. A. Mickiewicza 30, 30-059 Krakow, Poland*
[4] *Department of Applied Physics, Eindhoven University of Technology, 5600 MB Eindhoven, The Netherlands*
[5] *Netherlands Organisation for Applied Scientific Research (TNO), 2628 CK Delft, The Netherlands*
* These authors contributed equally.
† Sebastian.Heedt@Microsoft.com

SUPPLEMENTARY NOTE 1. FABRICATION DETAILS

Chips without Bottom Gates

Chips that contain devices with a global back-gate like the ones presented in Figs. 2 and 3 of the main text are fabricated on p^+-doped Si wafers covered with 285 nm of thermal SiO_2. The first fabrication step consists of patterning the bond pads via electron-beam lithography (EBL), W sputtering and lift-off in acetone. Afterwards, plasma-enhanced chemical vapour deposition (PECVD) of 600 nm of Si_3N_4 is performed followed by EBL, reactive-ion etching (RIE) with CHF_3/O_2 gases, resist lift-off and an oxygen plasma descum step to remove carbon residues. Eventually, nanowires are deposited under an optical microscope using a micromanipulator equipped with tungsten needles [1].

Chips with Bottom Gates

Chips with additional local bottom gates (used e.g. for the experiments in Figs. 4 and 5 of the main text) are fabricated by sputtering 17 nm of W on Si wafers covered with 285 nm of thermal SiO_x (protected by an Al_2O_3 etch-stop layer), followed by EBL patterning and RIE of the W layer with SF_6 gas. Next, 18 nm of a high-quality Al_2O_3 layer are deposited by atomic layer deposition (ALD), acting as the bottom-gate dielectric. Shadow walls on top of the bottom gates are created by first depositing 600 nm of Si_3N_4 by PECVD, followed by EBL patterning with precise alignment of the shadow walls with respect to the underlying fine bottom gates. Then, RIE with CHF_3/O_2 gases is used to selectively etch Si_3N_4 while the Al_2O_3 gate dielectric acts as an etch-stop layer. Finally, after the resist strip, an oxygen plasma descum step is used to remove carbon residues from the chips. The nanowires are then mechanically transferred on top of the bottom gates under an optical microscope using a micromanipulator equipped with tungsten needles [1].

Additional Fabrication Steps for N–S Devices

For devices with additional Ti/Au normal-metal contacts, such as the ones presented in Figs. 4 and 5 of the main text, an extra post-interface fabrication step is included. It consists of EBL patterning (solvents are removed from the resist via vacuum pumping instead of conventional resist baking to accommodate the low thermal budget), 40 s of argon ion milling at $1.5 \cdot 10^{-3}$ mbar with a commercial Kaufmann source in the load lock of an electron-beam evaporator, and in-situ evaporation of 10 nm/120 nm of Ti/Au at a pressure of $8 \cdot 10^{-8}$ mbar followed by lift-off in acetone. Note that this step is not strictly essential and could have been omitted. Bottom gates underneath the nanowire can open up the channels and tune the conductance. Combining this electrostatic gate control with additional Al contacts that are defined by shadow walls microns away from the N–S junction allows to entirely avoid post-interface fabrication for these devices.

Semiconductor Surface Treatment

To obtain a pristine, oxide-free semiconductor surface, we accomplish a gentle oxygen removal via atomic hydrogen radical cleaning. For this purpose, a custom-made H radical generator is installed in the load lock of our aluminium electron-gun evaporator. It consists of a gas inlet for H_2 molecules connected to a mass-flow controller and a tungsten filament at a temperature of about 1700 °C that dissociates a fraction of the molecules into hydrogen radicals [2].
The cleaning process is evaluated via the transport characteristics of InSb/Al nanowire Josephson junctions and TEM analysis of the same devices. In particular, we consider the magnitude of the supercurrents and the amount of interfacial oxide, measured by EDX, as critical indicators of the interface transparency. During optimization, we vary the process duration and the hydrogen flow, and keep the substrate temperature constant at 550 K. It has been demonstrated in the literature that this temperature results in an efficient cleaning of InSb, allowing for indium- and antimony-based oxides to be removed with similar efficiency [3, 4].
The optimal removal of the native oxide is achieved for a process duration of 30 mins and a hydrogen flow of 2 mln/min. During atomic hydrogen cleaning, the H_2 pressure is $6.3 \cdot 10^{-5}$ mbar. This recipe, which is used for all the devices shown in this paper, results in a constant EDX count of oxygen at the interface (i.e. the traces do not show oxygen peaks, see Fig. 2d of the main text) and yields the highest supercurrents in the Josephson junction devices ($\sim$ 90 nA).

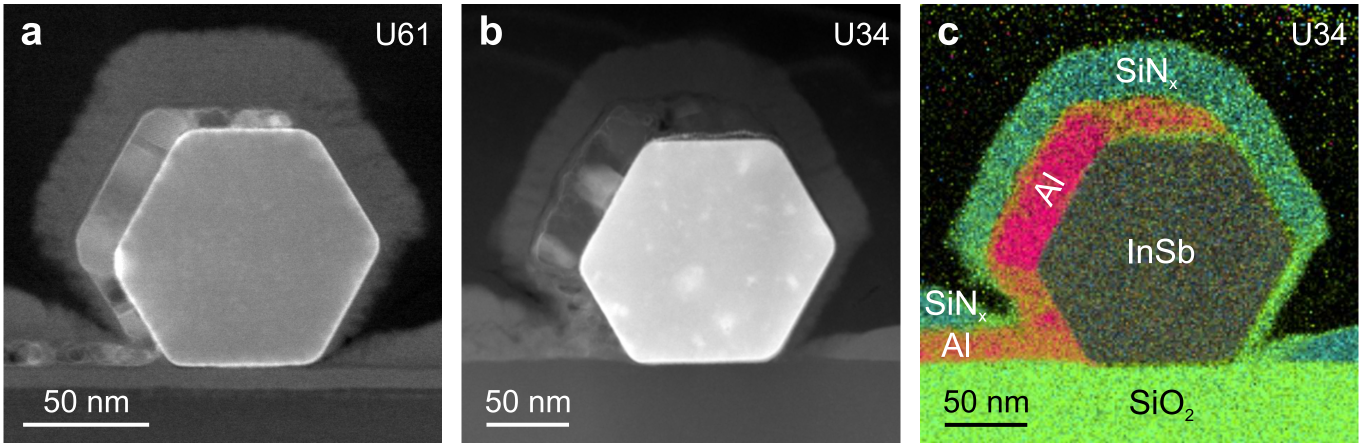

Supplementary Figure 1. Cross-sectional TEM images of InSb nanowires covered by a thin layer of Al. SiN_x was sputter-coated as a protective layer before focused ion beam (FIB) lamella preparation. **a** Annular dark-field (ADF) scanning TEM image of a nanowire cross-section. This sample is identical to the device presented in Figs. 2c–e of the main text (sample ID: U61). Al is deposited at an angle of 30° with respect to the substrate plane at $T = 140$ K. **b** ADF scanning TEM image of another nanowire cross-section (sample ID: U34). Here, 30 nm of Al are deposited at an angle of 25° with respect to the substrate plane at $T = 80$ K. **c** EDX elemental composite image of the device in panel (**b**) identifying the individual compounds and the Al thin film.

Superconductor Deposition

After cleaning the semiconductor surface, the chips are loaded into the main chamber of the evaporator and cooled down by actively circulating liquid nitrogen through the sample holder. After one hour of thermalization, aluminium is deposited by electron-beam evaporation at a typical rate of 0.2 nm/min.
The aluminium growth conditions are optimized by studying the quality of thin films deposited on Si substrates – typically also containing transferred InSb nanowires – at different evaporation rates, temperatures and angles. It is observed that evaporation angles close to 90° with respect to the substrate plane are favourable for aluminium thin-film growth, whereas at shallower angles the self-shadowing effect of Al atoms on the surface becomes more prominent, giving rise to columnar growth, possible voids in the film, and greater roughness [5, 6]. To minimize this angle-dependent self-shadowing effect, the substrate temperature can be slightly increased to give the atoms arriving at the substrate enough momentum to rearrange into a crystal before the next atoms arrive at the substrate. Our results and the work by Dong *et al.* [5] indicate that, for a fixed deposition rate, the temperature optimum depends on the evaporation angle.
In this work, a temperature optimum of around 140 K is found for Al growth at 30° with respect to the substrate plane, allowing for homogeneous 3-facet coverage of the hexagonal nanowires as well as a connection from the nanowires to the substrate. The Josephson junctions made at this growth temperature exhibit roughly four times

higher supercurrents than similar devices produced when Al was deposited at a substrate temperature of $\sim 80\,\mathrm{K}$. Cross-sectional TEM images of FIB lamellae from nanowires with Al grown at 140 K and 80 K are presented in Supplementary Figure 1a (as well as Figs. 2c,e of the main text) and Supplementary Figures 1b,c, respectively. Comparing these figures, the superior quality of the deposition at 140 K is evident; the nanowire facets are more uniformly covered and form a continuous film, the crystalline quality of the Al is higher and the oxidation of the Al facets is much less prominent than in the case of the deposition at 80 K (in Supplementary Figure 1c, the abundant oxide formation in the aluminium film at the top and bottom-left nanowire facets is especially noticeable).
In addition, Supplementary Figure 2 illustrates a comparison between a higher Al growth temperature (160 K) and Al grown at 140 K. The former results in both granular Al covering the middle nanowire facet, which is better observed in the tilt-view image in Supplementary Figure 2b, and a film on the substrate where the different grains are clearly distinguishable. In comparison, images corresponding to deposition at 140 K instead show a featureless Al film on the middle facet, where roughness is indiscernible under these SEM conditions (Supplementary Figure 2d), and a granular but more uniform Al structure on the substrate.

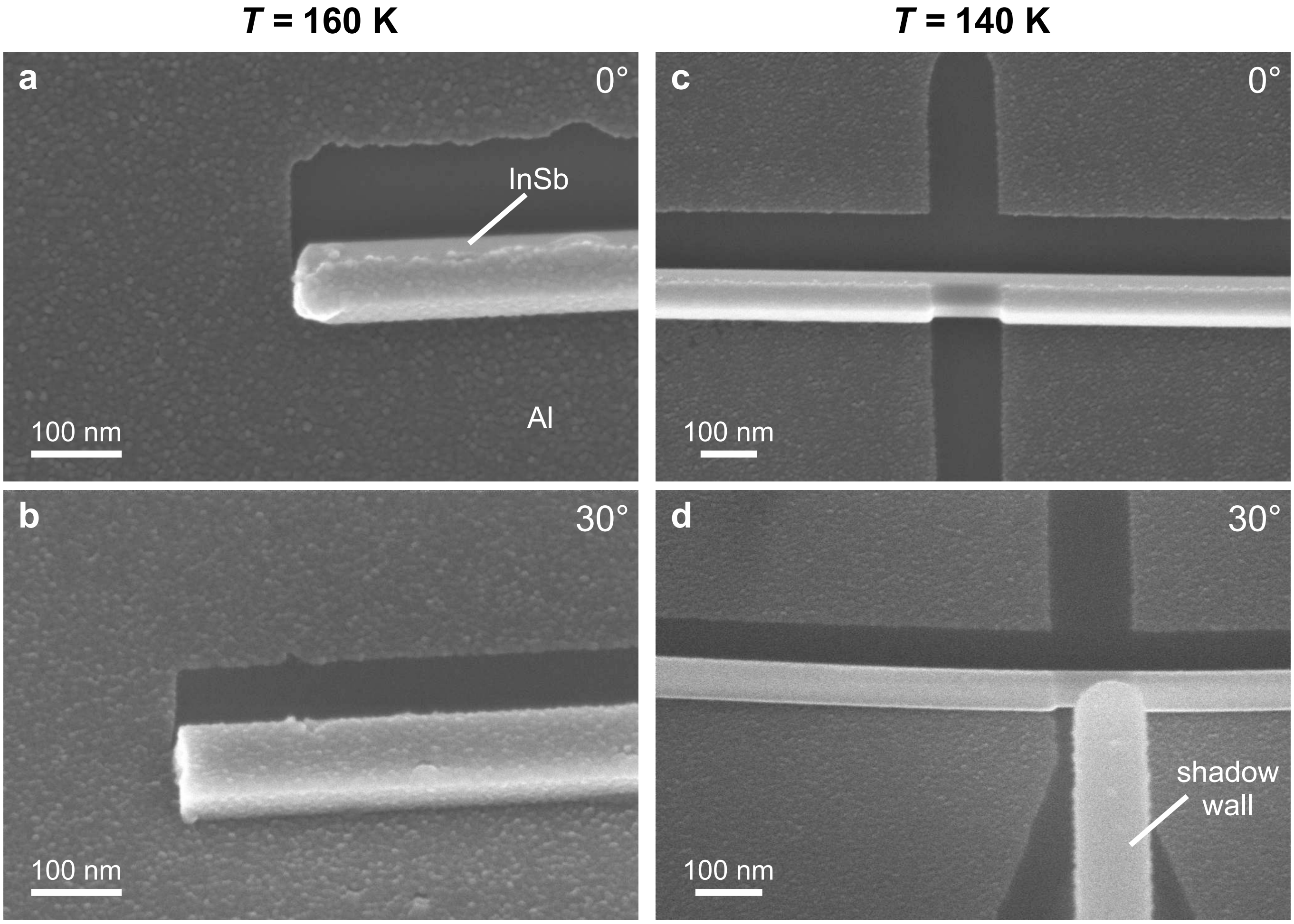

Supplementary Figure 2. SEM images of InSb nanowires with Al thin films deposited at different temperatures. For both samples the evaporation angle is 30° relative to the substrate plane. **a**, **b** Top-view and tilt-view (tilt angle: 30°) SEM images of InSb nanowires covered with an Al thin film deposited at 160 K. The maximum film thickness, which corresponds to the thin film on the middle nanowire facet, is 20 nm. **c**, **d** Top-view and tilt-view (tilt angle: 30°) SEM images of a nanowire Josephson junction. Here, the Al thin film is deposited at 140 K and the film thickness at the middle nanowire facet is 15 nm. Panel (**d**) exhibits a featureless Al shell on the middle wire facet, whereas grains are visible on the middle facet in the case of Al grown at 160 K (panel (**b**)).

Reproducibility and Typical Yield of Shadow-Wall Devices

As shown in Supplementary Figure 3, there are 4 unique orientations of a 3-facet Al shell on hexagonal nanowires. This rotational configuration has major implications for the electrostatics of the junctions, for the screening of the gates and it determines whether the electronic wave functions are pushed away from or towards the superconductor by the gates [7], renormalizing key parameters such as spin–orbit coupling, g factors and possibly gap hardness. To some degree, this random variation can be mitigated by post-selecting certain wire rotations after detailed SEM inspections, but it is very difficult to eliminate. Moreover, this would reduce the device yield by up to 75%. In contrast, the shadow-wall lithography method resolves this issue by removing any random variation in rotational configuration and enforcing the scenario in Supplementary Figure 3a.
Another source of random variation inherent in conventional fabrication methods is the design customization required for every device based on SEM images of individual nanowires. Shadow-wall lithography, however, relies on standardized designs and allows for convenient blind fabrication, which eliminates imaging and alignment steps. It offers inherently good alignment between the gates and the edges of the superconductors, resulting in low variability in device dimensions with many nearly identical devices made in a single deposition step.
By design, all nanowires are aligned along the same direction on the chip. This is an important bonus feature of the shadow-wall technique that ensures that the largest possible magnetic field of the vector magnet can be applied along every nanowire axis, which is critical for finding a topological phase transition.
As presented in Supplementary Note 2 for the Josephson junctions as well as in Supplementary Note 4 and Figs. 4 and 5 of the main text for the N–S devices, the magnitude of the induced gap is consistent across all devices. The atomic hydrogen cleaning yields pristine interfaces with high interface transparency resulting in hard-gap superconductivity on a par with state-of-the-art shadow-deposition methods. By omitting all or in some cases all but one fabrication steps, our approach avoids the ageing of the superconductor–semiconductor interface and yields many nominally identical devices.
Beyond the reduced variations among devices, a qualitatively new feature not offered by other techniques is the significant flexibility in device designs, such as the arbitrary shadow lengths and fundamentally new device geometries (e.g. 3-terminal Majorana devices or SQUIDs, see Supplementary Note 5).

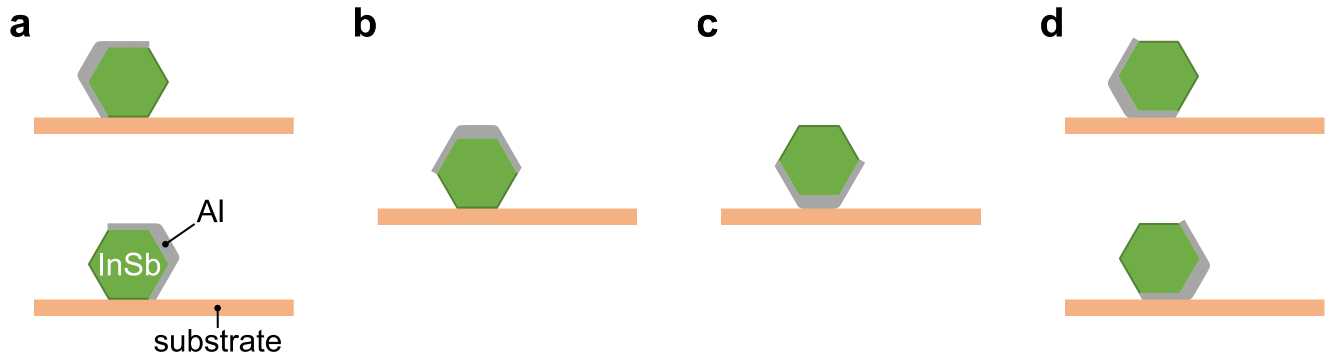

Supplementary Figure 3. The 4 unique orientations of the Al-covered facets on hexagonal nanowires.

Depending on the layout, our pre-patterned chips typically accommodate up to 16 nanowire devices. It is readily viable to have around 10 fully functional devices on a single chip to consistently optimize the fabrication parameters. The yield per chip can be affected by the accidental transfer of multiple wires at once or by nanowires breaking during the transfer. In Supplementary Figures 5, 6 and 7, we show scanning electron micrographs taken prior to the cool-down of the Josephson junctions. On the first chip (sample ID: U12) 13 nanowires are transferred and result in 12 working devices, i.e. where the junctions are well-defined. On the second chip (sample ID: U51) in total 12 nanowires are transferred, which yield 11 working devices. However, 2 turned out to be narrow nano-flakes [8, 9], which can be indistinguishable from nanowires in optical microscopy. On a third chip (sample ID: U55) 12 nanowires are positioned and yield 9 working devices. Among those, 7 are hexagonal-shaped nanowires and 2 turned out to be narrow nano-flakes. In Supplementary Figure 4, the reproducibility of the device dimensions within each chip and among two of the chips is illustrated by box plots of the extracted junction lengths.

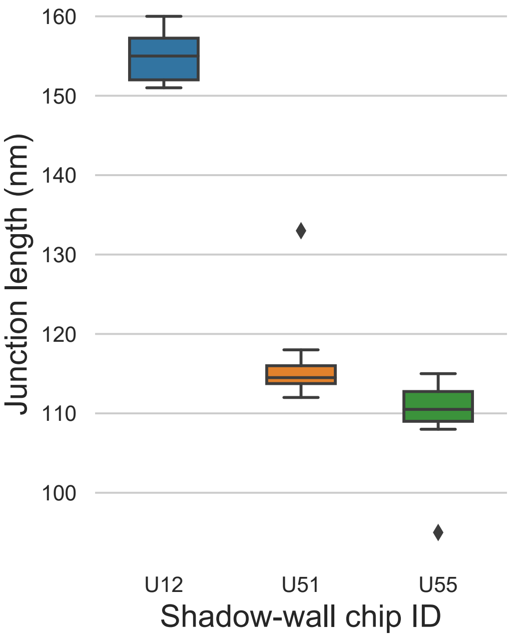

Supplementary Figure 4. Box plots showing the distribution of the Josephson junction length across the devices on chips U12 (Supplementary Figure 5), U51 (Supplementary Figure 6) and U55 (Supplementary Figure 7). The boxes denote the lower and upper quartiles of the distributions, the central horizontal lines denote the medians, and the whiskers show the minimum and maximum values of the junction length. The two diamonds are determined as outliers due to physical damage to the shadow walls. Chips U51 and U55 have been prepared using nominally the same shadow-wall layout and the median junction length differs by only 4 nm. For chip U12 the junction length is larger by design.

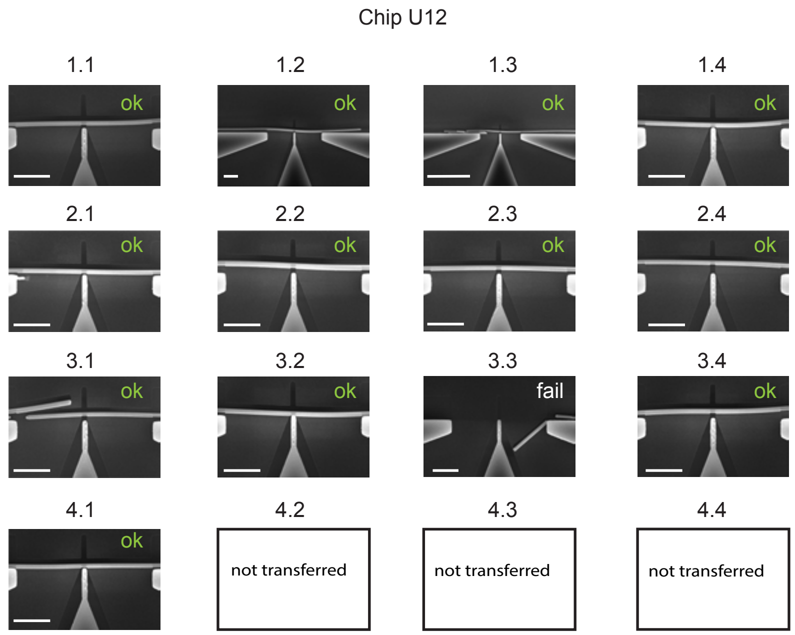

Supplementary Figure 5. Typical yield of the nanowire transfer for Josephson junction devices: Scanning electron micrographs of all Josephson junction devices on a typical chip (sample ID: U12) taken after the Al deposition. Out of 13 nanowire transfer attempts, 12 nanowires are perfectly positioned, and only in one case the transfer failed (device 3.3). The scale bars indicate 1 µm.

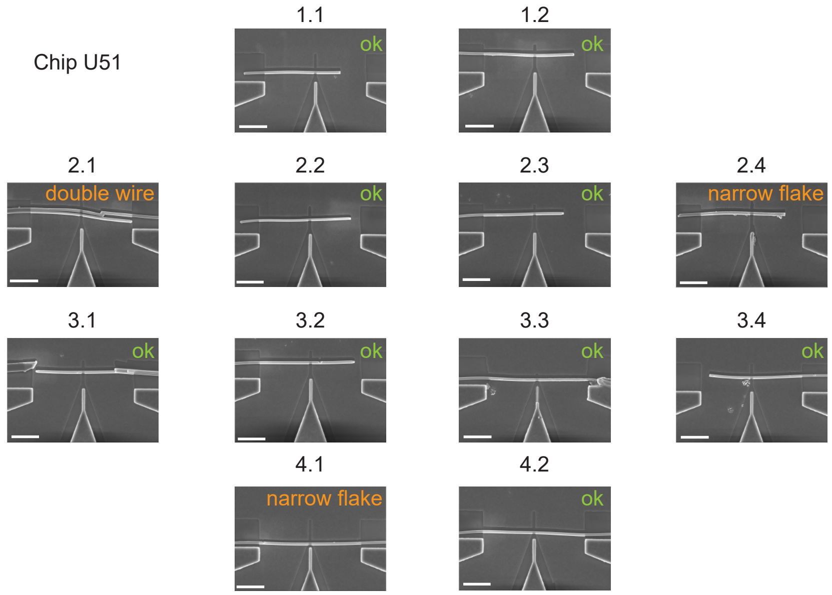

Supplementary Figure 6. Typical yield of the nanowire transfer for Josephson junction devices: Scanning electron micrographs of all Josephson junction devices on a typical chip (sample ID: U51) taken after the Al deposition. Out of 12 nanowire transfer attempts, 9 nanowires are perfectly positioned, 2 narrow flakes – rather than nanowires – are accidentally transferred (devices 2.4 and 4.1), and in one case, two nanowires are transferred in the same location (device 2.1). The scale bars indicate 1 µm.

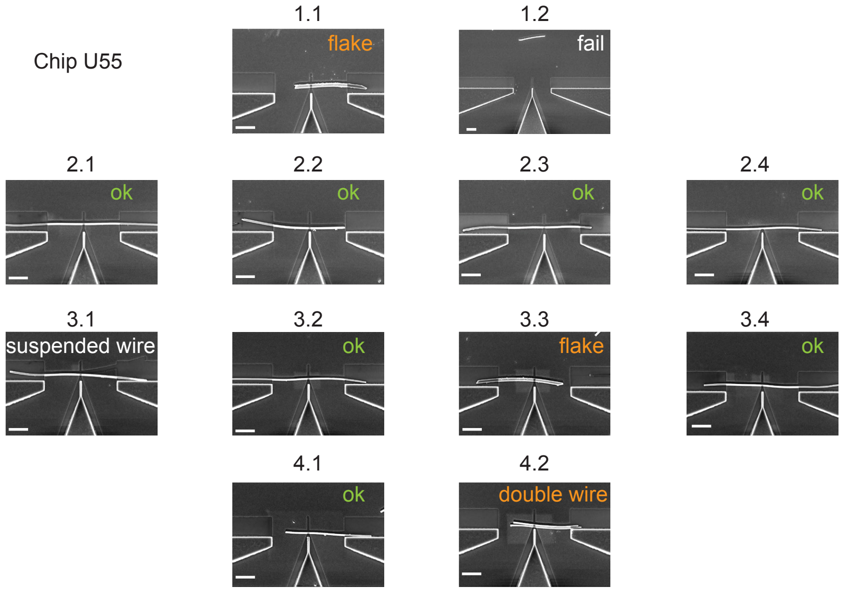

Supplementary Figure 7. Typical yield of the nanowire transfer for Josephson junction devices: Scanning electron micrographs of all Josephson junction devices on a typical chip (sample ID: U55) taken after the Al deposition. Out of 12 nanowire transfer attempts, 7 nanowires are perfectly positioned, 2 of them are not (devices 1.2 and 3.1), 2 narrow flakes – rather than nanowires – are accidentally transferred (devices 1.1 and 3.3), and in one case, two nanowires are transferred in the same location (device 4.2). The scale bars indicate 1 µm.

SUPPLEMENTARY NOTE 2. ADDITIONAL TRANSPORT MEASUREMENTS OF JOSEPHSON JUNCTIONS

In this section, we summarize the characteristics of the Josephson junction devices listed in Supplementary Table 1. All devices are fabricated by evaporating an Al thin film at an angle of 30° with respect to the substrate plane. Device 3 differs from the other samples in the thickness of the Al shell. We note that despite such a low shell thickness, all nanowire devices on sample U59 are in electrical contact with the Al film on the substrate.

Josephson junction	Sample ID/ device name	Evaporation angle	Channel width (nm)	Maximum Al thickness (nm)	Oxidation
device 1	U55/2.3	30°	100	16	in O_2 atmosphere
device 2	U51/1.2	30°	100	16	in O_2 atmosphere
device 3	U59/2.3	30°	100	11	Al_2O_3 capping
device 4	U55/3.3	30°	160	16	in O_2 atmosphere

Supplementary Table 1. Summary of the Josephson junction devices presented in this study. Devices 1, 2, and 3 are all nominally identical in their geometries with a nanowire diameter of 100 nm and a separation between the Al contacts of 115 nm. Device 3 was made with a thinner Al shell thickness and capped in situ with around 20 nm of Al_2O_3. Device 4 is a Josephson junction formed in an InSb nano-flake. Here, the channel width is 160 nm.

Device 1 The current and differential conductance in the normal state ($V_{SD} = 10\,mV$) display a steplike increase as a function of V_{BG} (Supplementary Figures 8a,b). The first two steps approximately align with the quantized values expected for one-dimensional transport, providing possible hints for ballistic transport at zero magnetic field. At lower bias voltage, features of the induced superconductivity appear such as the conductance peaks due to multiple Andreev reflections and the zero-bias supercurrent peak (Supplementary Figures 8c,d). A line-cut of Supplementary Figure 8c is presented in Fig. 3b of the main text, whereas a line-cut of Supplementary Figure 8d is shown in panel (e). Here, the experimental data (red trace) is fitted with the theoretical model (green trace) to identify the number and the transmissions of the nanowire subbands, which are plotted in Supplementary Figure 8f. Similarly, in Supplementary Figure 9a, we illustrate the extracted transmission probabilities of the three lowest subbands in the back-gate voltage range of Supplementary Figure 8c. The sum of these transmission probabilities extracted from the MAR pattern is compared to the normal-state conductance in Supplementary Figure 9b.

Device 2 The normal-state current and conductance ($V_{SD} = 10\,mV$) as a function of back-gate voltage are displayed in Supplementary Figures 10a,b. While conductance plateaus are more difficult to identify than in the case of device 1, the presence of an induced superconducting gap is clear from the MAR conductance peaks and the supercurrent peak (Supplementary Figures 10c,d). By fitting each line-cut of panel (c) (just like in panel (d)), we can extract the transmissions of the nanowire subbands across the full measurement range (Supplementary Figure 10e). The closing of the superconducting gap and the suppression of the switching current with the magnetic field aligned along three perpendicular orientations are shown in Supplementary Figure 11 and Supplementary Figure 12, respectively.

Device 3 Device 3 differs from the first two samples by having a significantly thinner Al shell. To protect the thin film from oxidation, the device is capped in situ with a 20 nm Al_2O_3 layer. This results in a large zero-field switching current of more than 50 nA (Supplementary Figure 13) and a critical magnetic field of $\sim 2\,T$ (Supplementary Figure 14).

Device 4 In this nano-flake device, the normal-state current manifests sharp steps and the differential conductance features quantized plateaus owing to ballistic transport in the junction (Supplementary Figures 15a,b). The presence of a moderate supercurrent (Supplementary Figure 15c) demonstrates that our fabrication technique can be used not only to proximitize one-dimensional nanowires, but also other types of nanostructures such as quasi-two-dimensional nano-flakes.

Voltage-biased conductance measurements in the tunnelling regime for 7 Josephson junctions (including devices 1–3) are depicted in Supplementary Figure 16. The average induced gap extracted for these devices is $\Delta_{ind} = 248 \pm 10\,\mu eV$. It is slightly higher than in the more transmissive regime at more positive gate voltages since the global back gate to some degree also tunes the coupling to the superconductor, in agreement with the literature [10].

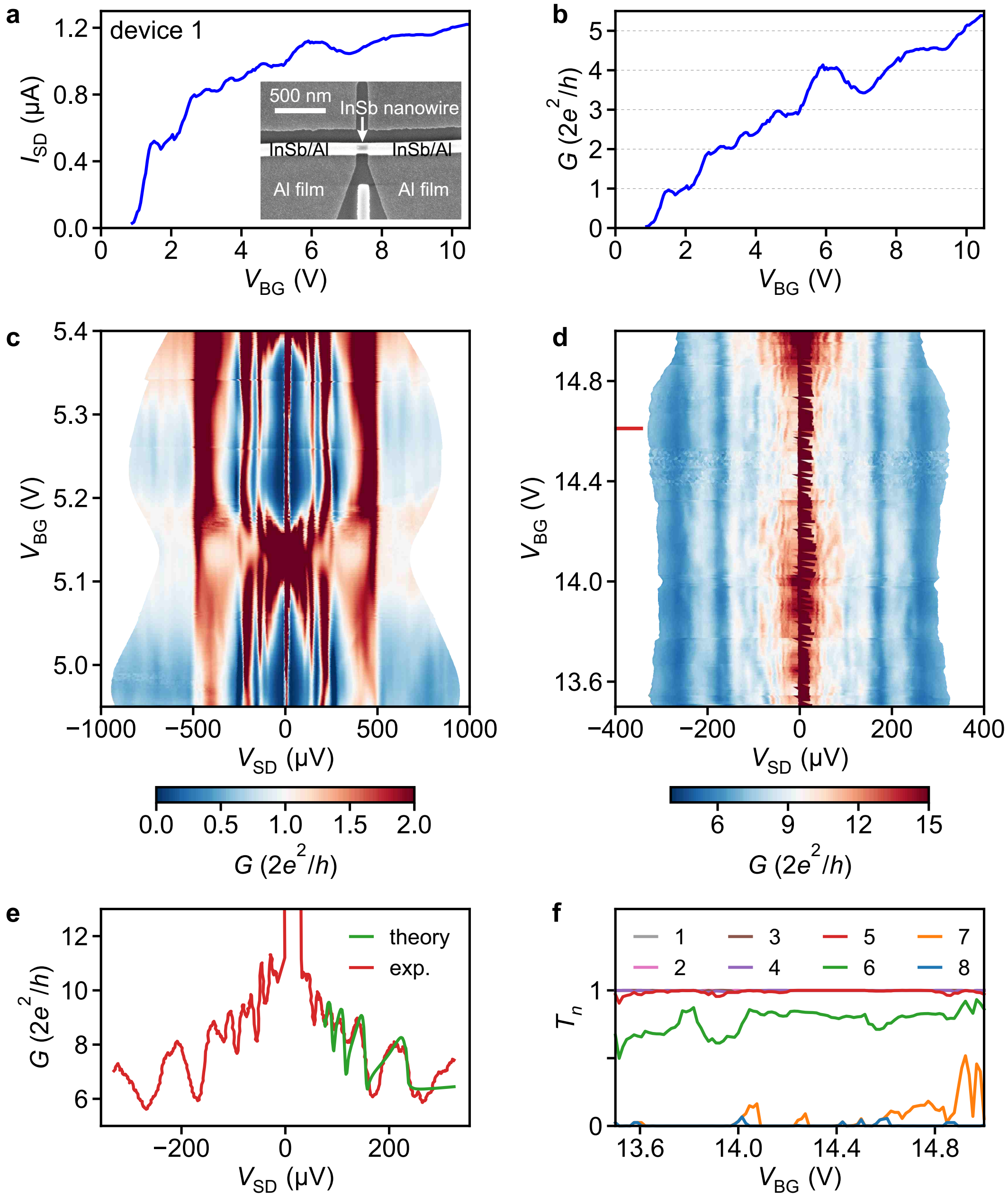

Supplementary Figure 8. Additional transport measurements of the first Josephson junction device. **a** I_{SD} vs. V_{BG} sweep at $V_{\mathrm{SD}} = 10\,\mathrm{mV}$, showing the field-effect tunability of the junction. Inset: scanning electron micrograph of the device. **b** DC conductance, G, after subtracting the series resistance of the setup, as a function of V_{BG} at 10 mV bias voltage. **c**, **d** G vs. V_{SD} and V_{BG} in the few-subbands and many-subbands regime, respectively: vertical features in both scans at constant bias voltages are the characteristic peaks originating from MARs. **e** Line-cut of (**d**) at $V_{\mathrm{BG}} = 14.61\,\mathrm{V}$ in red and best fit of the trace in green according to the coherent scattering model in Supplementary Note 3. **f** Extracted transmission probabilities, T_n, as a function of V_{BG} in the multi-subbands regime, with $n \in \{1, 2, \ldots, 8\}$. In this back-gate voltage range, the transmission of the first five subbands is already saturated at $T_n = 1$.

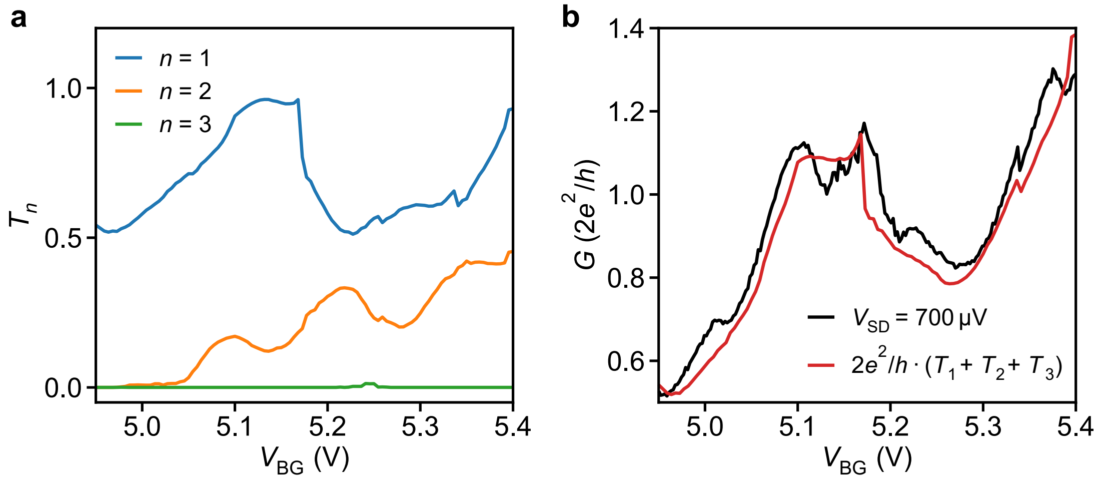

Supplementary Figure 9. Tunability of the subbands of the first Josephson junction device. **a** Transmission probabilities, T_n, of the first three subbands as a function of V_{BG}. The parameters are extracted by fitting the conductance map of Supplementary Figure 8c with the coherent scattering model described in Supplementary Note 3. **b** Out-of-gap conductance as a function of V_{BG} in black (i.e. vertical line-cut of Supplementary Figure 8c at $V_{\mathrm{SD}} = 700\,\mu\mathrm{V}$) together with the sum of the transmission probabilities in red.

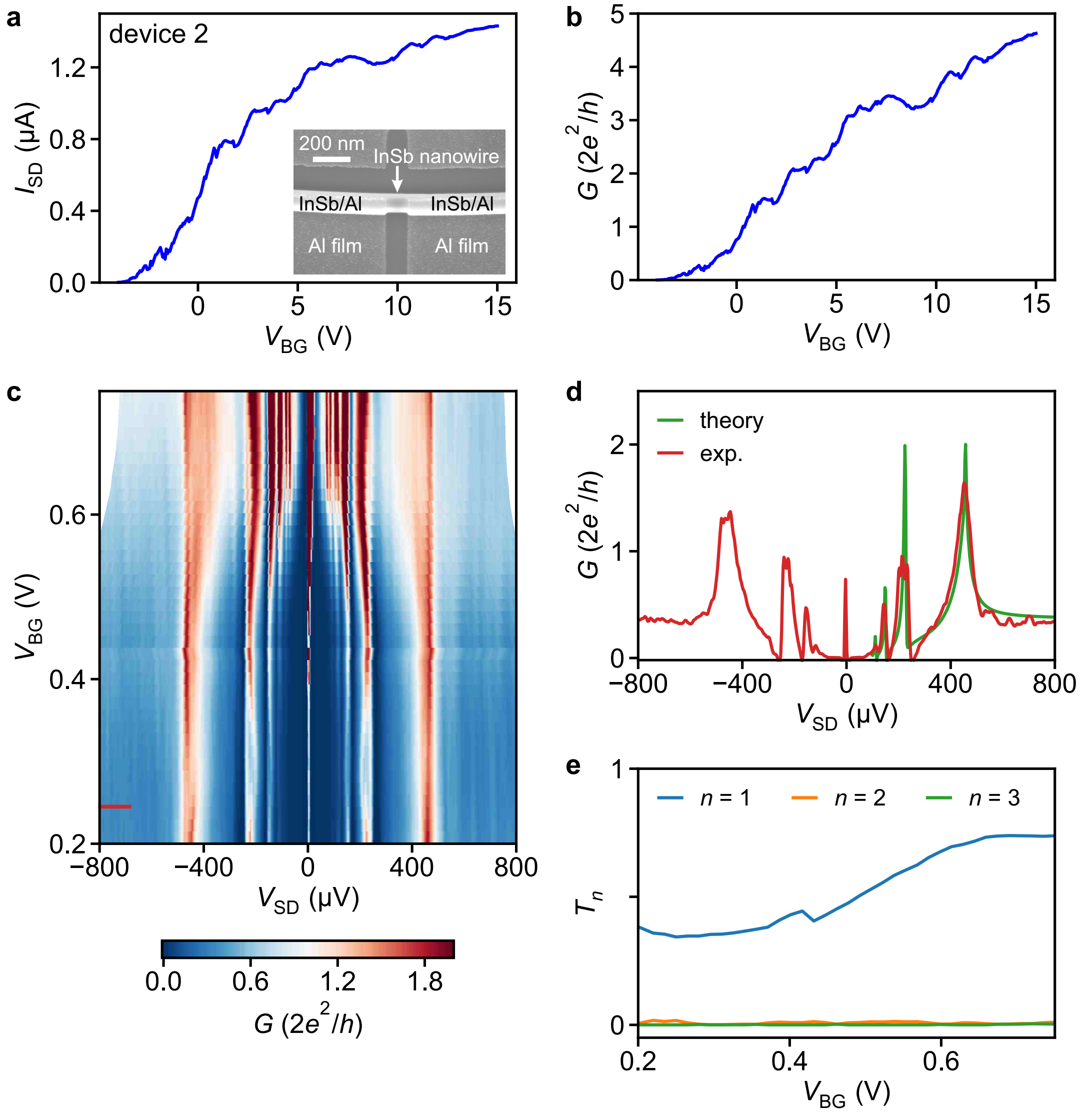

Supplementary Figure 10. Additional transport measurements of the second Josephson junction device. **a** I_{SD} vs. V_{BG} sweep at $V_{\mathrm{SD}} = 10\,\mathrm{mV}$, showing the field-effect tunability of the junction. Inset: scanning electron micrograph of the device. **b** DC conductance, G, after subtracting the series resistance of the setup, as a function of V_{BG} at $10\,\mathrm{mV}$ bias voltage. **c** G vs. V_{SD} and V_{BG} in the weak-tunnelling regime: subharmonic gap features correspond to different orders of MARs. **d** Line-cut of (**c**) at $V_{\mathrm{BG}} = 0.25\,\mathrm{V}$ in red and best fit of the trace in green according to the coherent scattering model in Supplementary Note 3. **e** Extracted transmission probabilities, T_n, depicted as a function of V_{BG} with $n \in \{1, 2, 3\}$.

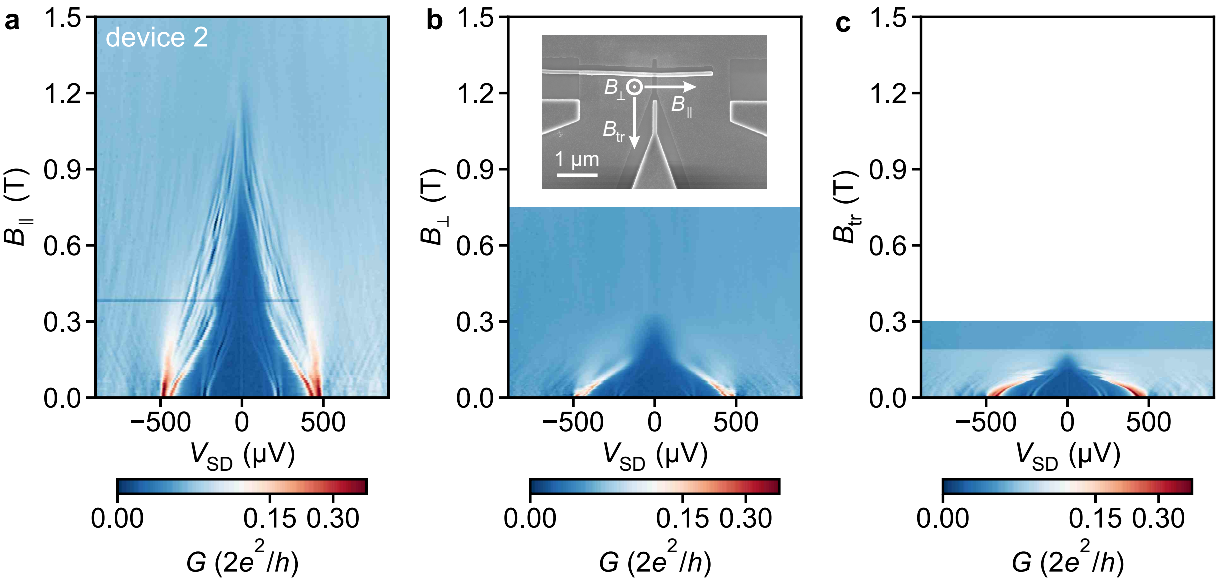

Supplementary Figure 11. Superconducting critical magnetic fields of the second Josephson junction device. Colour maps of G vs. V_{SD} and magnetic field taken at $V_{\mathrm{BG}} = 1.45\,\mathrm{V}$ for different magnetic field orientations: in **a** the field $B_{\|}$ is oriented parallel to the nanowire direction, in **b** $B_{\perp}$ is orthogonal to the plane of the substrate, and in **c** the transversal field B_{tr} is orthogonal to the nanowire direction but in the substrate plane. The inset in panel (**b**) shows a scanning electron micrograph of the device together with the different magnetic field directions.

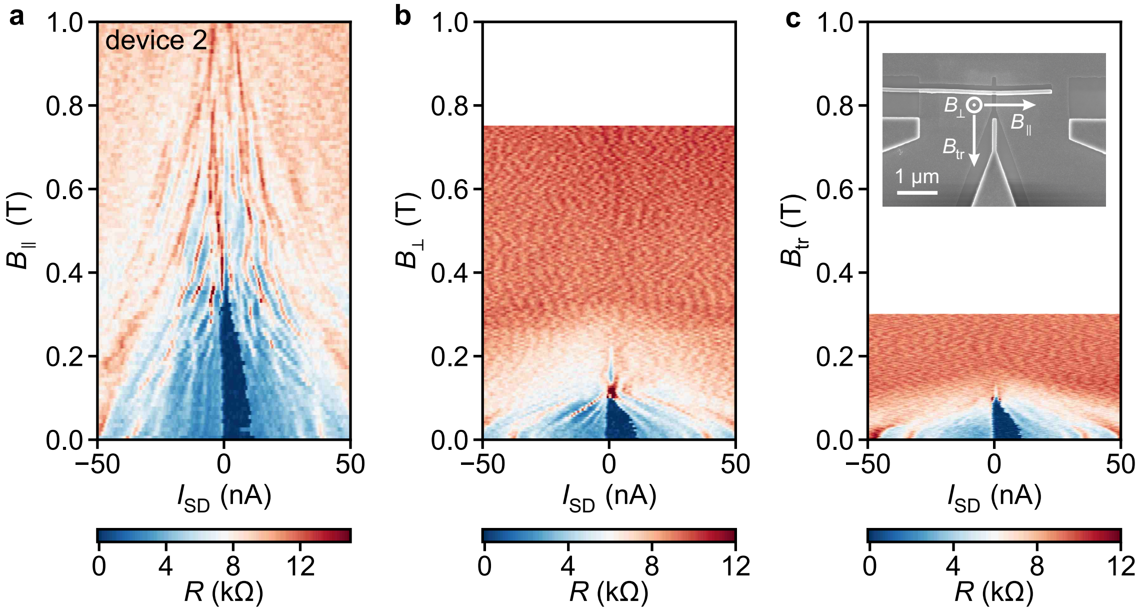

Supplementary Figure 12. Switching current of the second Josephson junction device in the open-channel regime ($V_{\mathrm{BG}} = 5.7\,\mathrm{V}$). Differential resistance, R, as a function of I_{SD} and magnetic field in three different orientations: **a** Magnetic field, $B_{\|}$, aligned parallel to the nanowire, **b** magnetic field, $B_{\perp}$, oriented out-of-plane, and **c** transversal in-plane magnetic field, B_{tr}. The vectors in the inset of panel (**c**) illustrate the three field orientations.

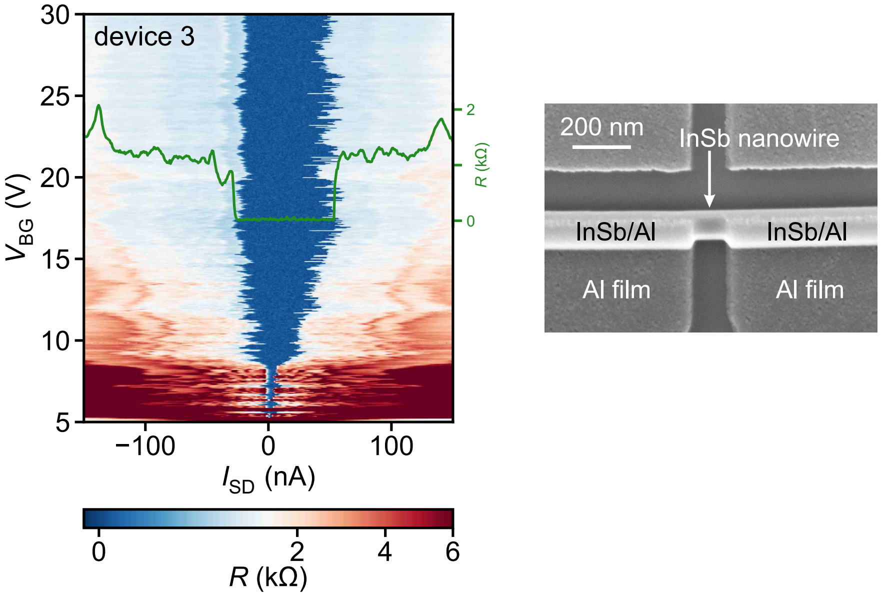

Supplementary Figure 13. Back-gate dependence of the switching current of the third Josephson junction device. Colour map of R as a function of I_{SD} and V_{BG}; the green trace is taken at $V_{BG} = 13.82\,V$. The switching current (in dark blue) is suppressed in the low back-gate voltage regime. The inset on the right shows a scanning electron micrograph of the device. The Al segments are capped with a protective layer of Al_2O_3 evaporated at an angle of $30°$.

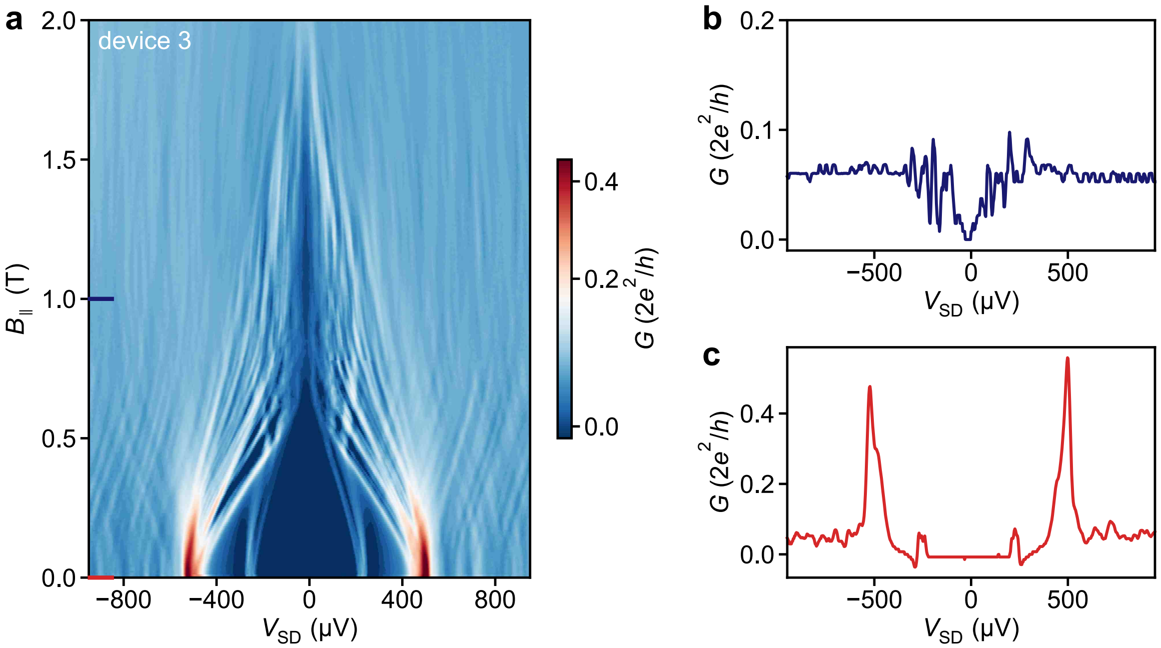

Supplementary Figure 14. Transport measurements of the third Josephson junction device. **a** V_{SD} vs. $B_\parallel$ in the tunnelling regime. Owing to the thinner Al shell, the superconducting critical field is $B_c \sim 2\,T$, much larger than for the previous two junctions. The tunnelling conductance peaks at $\pm 2\Delta_{ind}$ split into a manifold of resonances at a finite magnetic field due to the different g factors of the discrete quasiparticle states at the gap edge. **b**, **c** Line-cuts of (**a**) at the positions indicated by the two lines.

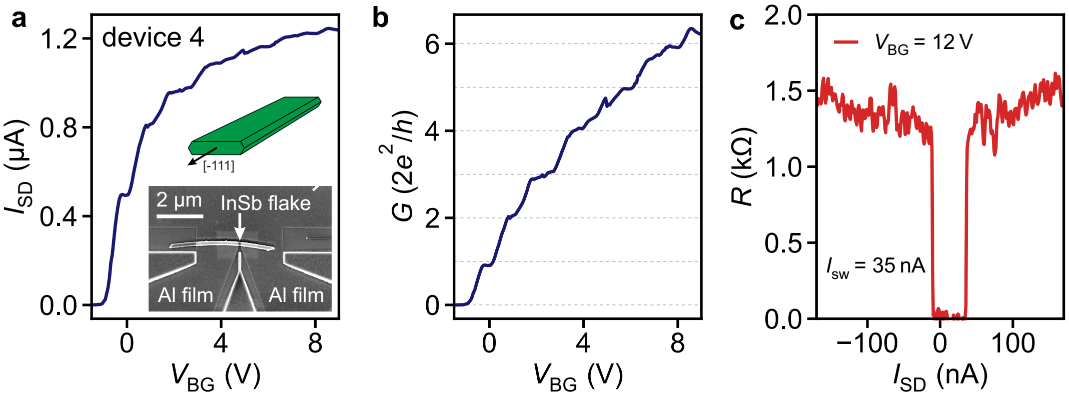

Supplementary Figure 15. Ballistic transport and supercurrent in an InSb flake Josephson junction (device 4) at zero magnetic field. **a** I_{SD} vs. V_{BG} at $V_{\mathrm{SD}} = 10\,\mathrm{mV}$. Bottom inset: scanning electron micrograph of the nano-flake Josephson junction. Top inset: schematic of the cross-section of the nano-flake [9]. **b** G vs. V_{BG} at $V_{\mathrm{SD}} = 10\,\mathrm{mV}$. The conductance displays distinct plateaus at multiples of $2e^2/h$, indicating the stepwise population of the one-dimensional subbands in the nanowire. **c** R vs. I_{SD} at $V_{\mathrm{BG}} = 12\,\mathrm{V}$. The device exhibits a switching current of $\sim 35\,\mathrm{nA}$ in the open-channel regime.

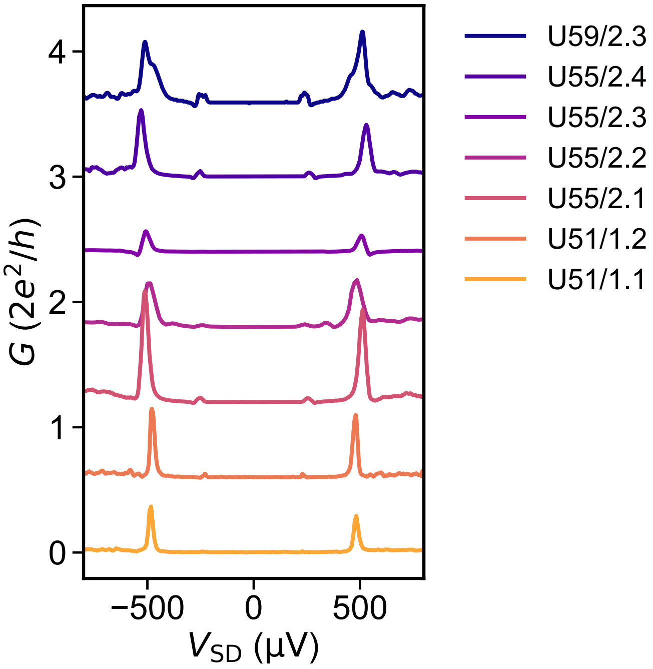

Supplementary Figure 16. Voltage-biased induced gap measurements on 7 different Josephson junction devices in the tunnelling regime (cf. Supplementary Table 1). Neighbouring traces are offset in conductance by $0.6 \cdot 2e^2/h$ for clarity. The average nanowire diameter of these Josephson junctions is $99 \pm 5\,\mathrm{nm}$. The average induced gap for these devices of $\Delta_{\mathrm{ind}} = 248 \pm 10\,\mathrm{\mu eV}$ is extracted from the best fits of these traces according to the coherent scattering model in Supplementary Note 3 and it corresponds to the conductance peaks where the coherence peaks on both sides of the junctions are aligned.

SUPPLEMENTARY NOTE 3. MODELLING OF ANDREEV TRANSPORT

Modelling of the Conductance of a Biased Josephson Junction and the Fitting Procedure

We calculate the conductance of a voltage-biased Josephson junction following the approach of ref. [11]. In the model, we account for the electrons and holes propagating through the normal region of the junction with the transparency T. The quasiparticles are accelerated by the voltage V_{SD} applied to the structure and are Andreev reflected at the superconducting leads with the induced superconducting gap Δ_{ind}. The sequential Andreev reflections imprint the conductance with the subgap features appearing at $V_{\mathrm{SD}} = 2\Delta_{\mathrm{ind}}/Ne$, where N is integer – see Supplementary Figure 17.

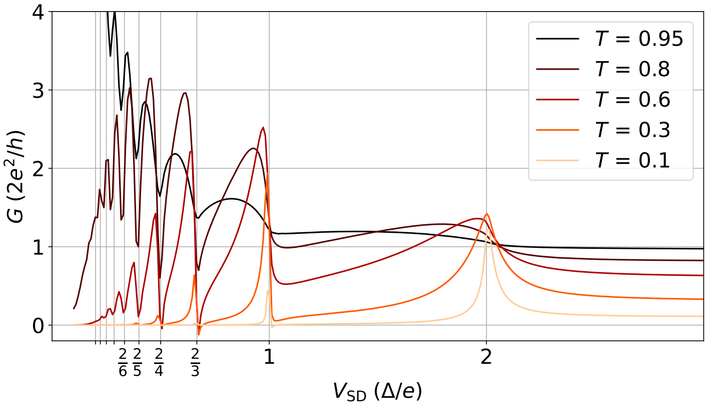

Supplementary Figure 17. Conductance, G, of a single-mode Josephson junction versus bias voltage, V_{SD}, for five transparencies (T) of the normal region.

For the analysis of the experimental conductance traces, we estimate the total conductance $G_{\mathrm{theory}}(V_{\mathrm{SD}})$ of a multimode nanowire Josephson junction as a sum of M single-mode contributions resulting from the presence of M modes of the transverse quantization [12]:

$$G_{\mathrm{theory}}\left(V_{\mathrm{SD}}\right) = \sum_{i=1}^{M} G_i\left(V_{\mathrm{SD}}, T_i, \Delta_{\mathrm{ind}}\right), \tag{1}$$

where T_i is the transmission probability for the i-th mode.
We obtain T_i and Δ_{ind} (induced in the nanowire by the presence of the Al shell) by fitting the numerically calculated conductance to the experimental one by minimizing $\chi = \int [G_{\mathrm{exp}}(V_{\mathrm{SD}}) - G_{\mathrm{theory}}(V_{\mathrm{SD}})]^2 dV_{\mathrm{SD}}$. M is a free parameter of the fitting procedure and it is chosen as the smallest number for which at least one of the parameters T_i is zero.

Theory for Multiple Andreev Reflections in the Presence of Subgap States

The original theory developed in ref. [11] assumes a bulk superconducting density of states in the leads. To account for different properties of the two leads, especially the presence of subgap states in one of the contacts, we extend this theory as follows.
We consider a Josephson junction consisting of two superconducting electrodes connected through a normal scattering region. We assume that the first contact is kept at zero voltage, while the second one is biased at V_{SD}.
In the normal region, adjacent to the L-th lead, the quasiparticle wave function takes the form,

$$\Psi_L = \sum_n \left[\begin{pmatrix} A_n^L \\ B_n^L \end{pmatrix} e^{ikx} + \begin{pmatrix} C_n^L \\ D_n^L \end{pmatrix} e^{-ikx} \right] e^{-i[E+neV_{\mathrm{SD}}]t/\hbar}, \tag{2}$$

where A_n^L, C_n^L (B_n^L, D_n^L) correspond to the electron (hole) amplitudes, the time dependence stems from the voltage applied to the leads and x points in the direction opposite to the scattering region.
We describe the scattering properties of the normal region by the scattering matrix:

$$S_0 = \begin{pmatrix} r & t \\ t & -r \end{pmatrix}, \tag{3}$$

which sets the transmission probability through the scattering region with the transmission amplitude $t = \sqrt{T}$ and the reflection amplitude $r = \sqrt{1-T}$.
We rely on the short-junction approximation and use the energy-independent S_0 to setup the matching conditions for the wave functions Ψ_L. The electron and hole coefficients are related by:

$$\begin{pmatrix} A_n^1 \\ A_{n+1}^2 \end{pmatrix} = S_0 \begin{pmatrix} C_n^1 \\ C_{n+1}^2 \end{pmatrix}, \tag{4}$$

and

$$\begin{pmatrix} D_n^1 \\ D_{n-1}^2 \end{pmatrix} = S_0^* \begin{pmatrix} B_n^1 \\ B_{n-1}^2 \end{pmatrix}, \tag{5}$$

respectively. The shifts of the indexes correspond to the changes of quasiparticle energies due to the bias voltage.
At each superconductor–normal-conductor interface we take into account the Andreev reflection:

$$\begin{pmatrix} C_n^L \\ B_n^L \end{pmatrix} = \begin{pmatrix} a_n & 0 \\ 0 & a_n \end{pmatrix} \begin{pmatrix} D_n^L \\ A_n^L \end{pmatrix}, \tag{6}$$

with the amplitude $a_n \equiv a(E + neV_{\mathrm{SD}})$, where,

$$a(E) = \frac{1-\delta_{L,1}\Gamma(E)}{\Delta_{\mathrm{ind}}} \begin{cases} E - \mathrm{sgn}(E)\sqrt{E^2 - \Delta_{\mathrm{ind}}^2} & |E| > \Delta_{\mathrm{ind}} \\ E - i\sqrt{\Delta_{\mathrm{ind}}^2 - E^2} & |E| \leq \Delta_{\mathrm{ind}}. \end{cases} \tag{7}$$

The Andreev reflection amplitude is modified by the factor $[1 - \delta_{L,1}\Gamma(E)]$ where,

$$\Gamma(E) = \frac{\gamma^2}{(E \pm E_0)^2 + \gamma^2}, \tag{8}$$

is the Lorentzian distribution that accounts for absorption of the quasiparticles in the subgap states (with the energy $\pm E_0$) in the first lead. We set $\gamma = 4\,\mu\mathrm{eV}$.
The electronic excitations in the normal part of the junction originate from the quasiparticles incoming from the nearby superconducting contacts. We therefore write down equation (6) including the quasiparticle source terms [13]:

$$\begin{aligned} \begin{pmatrix} C_n^L \\ B_n^L \end{pmatrix} &= \begin{pmatrix} a_n & 0 \\ 0 & a_n \end{pmatrix} \begin{pmatrix} D_n^L \\ A_n^L \end{pmatrix} \\ &+ \begin{pmatrix} J(E + eV_L) \\ 0 \end{pmatrix} \frac{1}{\sqrt{2}} \delta_{p,e}\delta_{s,L}\kappa_L^+ \\ &+ \begin{pmatrix} 0 \\ J(E - eV_L) \end{pmatrix} \frac{1}{\sqrt{2}} \delta_{p,h}\delta_{s,L}\kappa_L^-, \end{aligned} \tag{9}$$

with $J(E) = \sqrt{[1 - a(E)^2]F_D(E)}$, where $F_D(E, T = 30\ \mathrm{mK})$ is the Fermi distribution. In equation (9) p sets the injected quasiparticle type, s determines the lead in which we consider the source term, $\kappa_1^\pm = \delta_{n,0}$, $\kappa_2^\pm = \delta_{n,\pm 1}$ keep track of the quasiparticle energy shifts due to the bias, and where $V_1 = 0$ and $V_2 = V_{\mathrm{SD}}$.
We calculate the current I^L in the L-th lead as:

$$I^L = \sum_{\imath=-I_{\max}}^{I_{\max}} I_\imath^L e^{\imath V_{\mathrm{SD}} e i t/\hbar}, \tag{10}$$

with the Fourier components,

$$I_\imath^L = \frac{e}{\hbar\pi} \sum_{s=1,2} \sum_{p=e,h} \int_{-\infty}^{\infty} dE \sum_{n=-N_{\max}}^{N_{\max}} (\mathbf{U}_{\imath+n}^{L*}\mathbf{U}_n^L - \mathbf{V}_{\imath+n}^{L*}\mathbf{V}_n^L). \tag{11}$$

$\mathbf{U}_n^L = \left(A_n^L, B_n^L\right)^T$ and $\mathbf{V}_n^L = \left(C_n^L, D_n^L\right)^T$ are vectors that consist of the electron and hole amplitudes. The DC current is obtained for $\imath = 0$ and subsequently used to calculate the conductance, $G = dI^L/dV_{\mathrm{SD}}$. To efficiently sample the non-uniform conductance trace we use the Adaptive package [14].

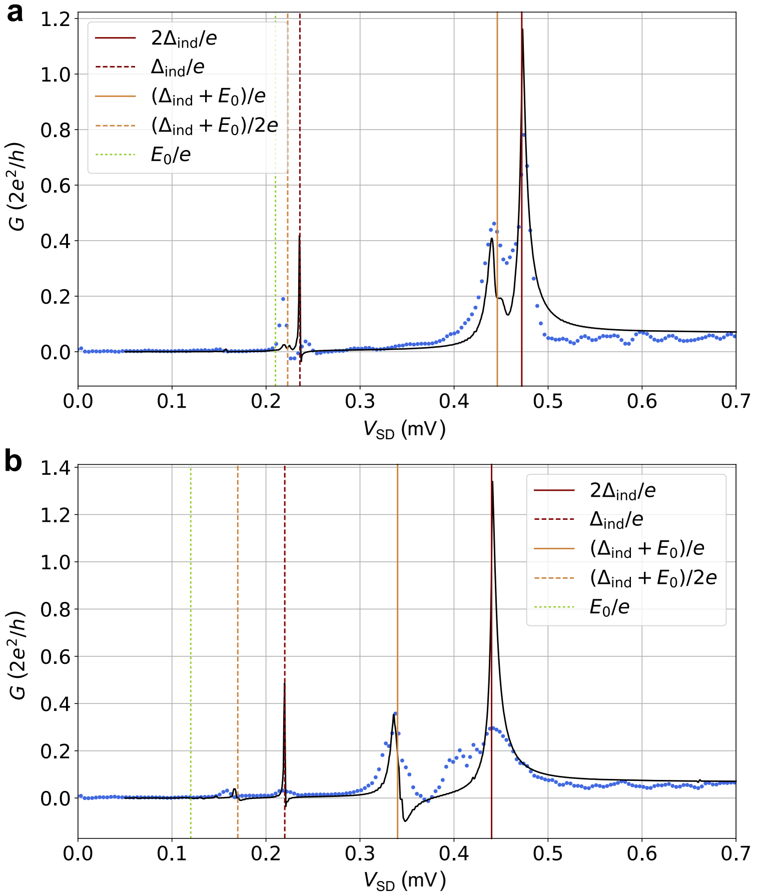

Supplementary Figure 18. Experimental (blue dots) and theoretical (black curves) conductance traces of a Josephson junction with a subgap state in one of the superconducting leads. **a** is for $B_{||} = 0$ and **b** is for $B_{||} = 0.2\,\mathrm{T}$.

In Supplementary Figure 18, we show the calculated MAR conductance traces (black curves) together with two cross-sections (blue dots) from the experimental map in Fig. 3c of the main text. We focus here on two cases: $B_{||} = 0$ and $B_{||} = 0.2\,\mathrm{T}$ with the parameters used for the calculations given in the first and second row of Supplementary Table **2**, respectively. The calculated traces agree qualitatively well with the data: they capture the peak positions and the overall line shape. In particular, we observe two ordinary MAR peaks at $V_{\mathrm{SD}} = 2\Delta_{\mathrm{ind}}/Ne$ with $N = 1, 2$ and two peaks induced by the presence of the subgap state at $V_{\mathrm{SD}} = (\Delta_{\mathrm{ind}} + E_0)/Ne$ with $N = 1, 2$. The increase of the magnetic field significantly alters the energy of the subgap state causing a further splitting between the MAR and the subgap-induced peaks.

$\boldsymbol{B_{\|\|}}$ (T)	$\boldsymbol{\Delta_{\mathrm{ind}}}$ **(μeV)**	$\boldsymbol{T_1}$	$\boldsymbol{E_0}$ **(μeV)**
0	236	0.065	210
0.2	220	0.065	120

Supplementary Table 2. Parameters used for the calculation of the conductance traces in Supplementary Figure 18.

To better understand the transport features in Fig. 3c of the main text, we simulate the conductance assuming a single subgap state whose energy evolves linearly in the magnetic field as $E_0 = \pm(E_{B_\parallel=0} - \frac{1}{2}g\mu_B B_\parallel)$, where g = 18 and $E_{B_\parallel=0} = 210\,\mu$eV. The result is shown in Supplementary Figure 19a, where we have assumed a junction transmission of $T_1 = 0.065$ and a magnetic field dependence of the gap given by $\Delta_{ind} = \Delta_0(1 - B_\parallel^2/B_c^2)$ [15, 16] with $B_c = 1.1$ T and $\Delta_0 = 236\,\mu$eV. In Supplementary Figures 19b–d, we illustrate the quasiparticle transport processes for different magnetic fields. The conductance peaks at $V_{SD} = \pm 2\Delta_{ind}/e$ correspond to an energy difference of $2\Delta_{ind}$ as denoted by the red arrows. If the electron transfer involves a subgap state at energy E_0 on one side of the junction, the corresponding bias voltage is $V_{SD} = (\Delta_{ind} + E_0)/e$ (Supplementary Figure 19d). As the magnetic field is increased, the subgap state moves to lower energies. Once the state is at zero energy ($E_0 = 0$), electrons only require an energy of $eV_{SD} = \Delta_{ind}$ (Supplementary Figure 19c). In Fig. 3c of the main text, this occurs around $B_\parallel = 0.4$ T. As the subgap state crosses zero energy, electrons again require an energy of $eV_{SD} = \Delta_{ind} + E_0$ to cross the junction via this state (Supplementary Figure 19b). If the junction is more transmissive, as is the case for Fig. 3c of the main text, also a MAR process occurs, identified by the conductance peak that emerges at $V_{SD} \sim 0.24$ mV at zero field and is associated with an energy Δ_{ind}. In addition, when the subgap state moves to lower energies due to the Zeeman effect, it also allows for a MAR process to occur, which results in a splitting of the MAR peak. With increasing magnetic field, the superconducting gap on both sides of the junction shrinks, resulting in the scenario shown in Supplementary Figure 19a, where the subgap state moves as a function of $B_\parallel$ from $V_{SD} = 2\Delta_{ind}/e$ down to Δ_{ind}/e and back up to $2\Delta_{ind}/e$. Considering that multiple subgap states can peel off from the gap edge with different associated g factors, a rich and complex pattern can occur. This interpretation of the involved transport processes is supported by the numerical simulation.

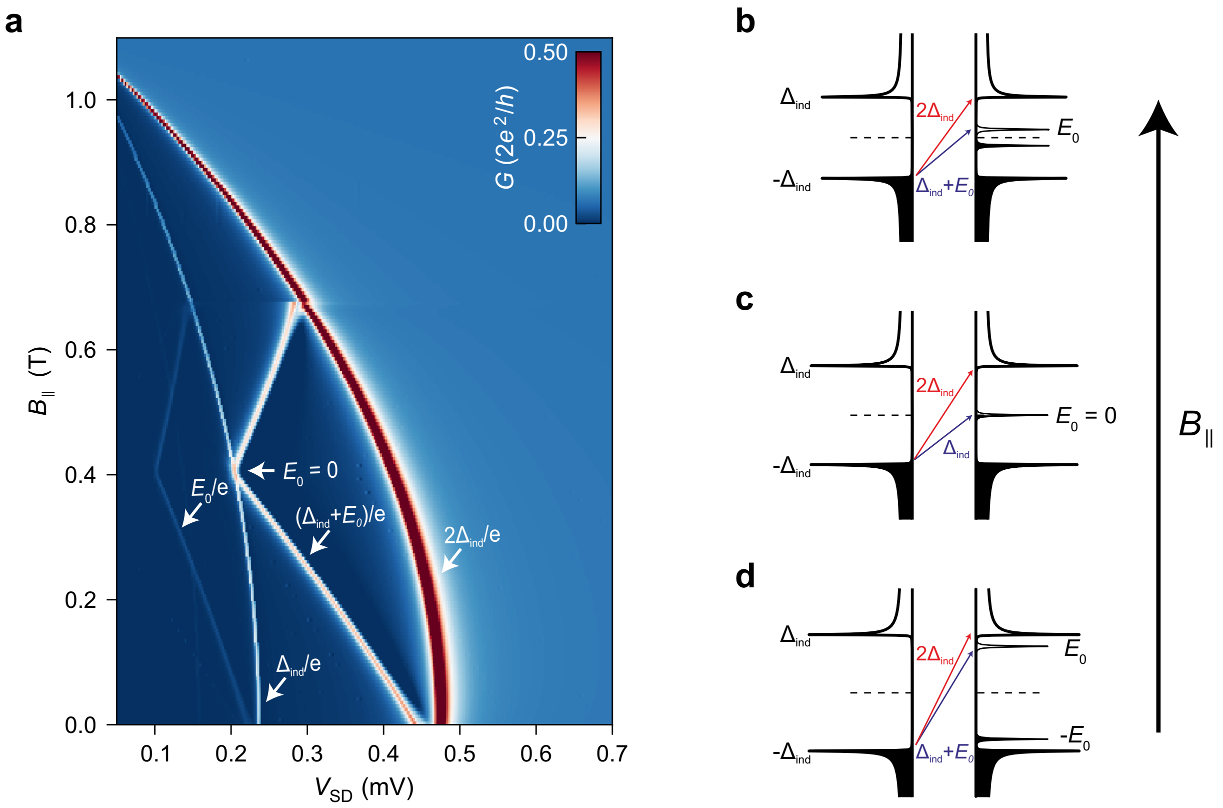

Supplementary Figure 19. Multiple Andreev reflections in the presence of a subgap state. **a** Calculated conductance in the presence of a subgap state at energy E_0. Both quantities Δ_{ind} and E_0 vary with magnetic field. **b**–**d** Schematics of the first-order multiple Andreev reflection processes for different magnetic fields increasing from the bottom to the top panel. The superconducting gap is varied accordingly.

SUPPLEMENTARY NOTE 4. ADDITIONAL TRANSPORT MEASUREMENTS IN NORMAL METAL/SUPERCONDUCTOR JUNCTIONS

Calibrating the AC conductance

The AC conductance is measured using a standard low-frequency lock-in technique. Some of the employed current-to-voltage amplifiers have been found to suffer from a relatively low bandwidth. This required a recalibration of the measured differential conductance of the N–S devices. The approach shown here is similar to a calibration procedure developed by Jouri Bommer, Guanzhong Wang and Michiel de Moor (see also guidelines on lock-in measurements by the same authors: http://homepage.tudelft.nl/q40r9/lockin-meas-guide-v20200603.pdf).
Supplementary Figure 20 shows the raw conductance data from Fig. 4 of the main text prior to the subtraction of any series resistance. For the mapping of the lock-in conductance, $G_{\rm LI}$, to the numerical DC conductance, $G_{\rm num}$, the data is binned into a two-dimensional histogram (resolution $0.003 \cdot 2e^2/h$). Since the numerical conductance suffers from noise, we determine the centre of the distribution for each bin of $G_{\rm LI}$ by fitting a Gaussian distribution to the histogram of $G_{\rm num}$ (see right panel of Supplementary Figure 20). Data points that are more than 5 standard deviations from the centre of the distributions are discarded as outliers. Here, the mapping yields the parametrization $G_{\rm num} = -0.016 \cdot G_{\rm LI}^2 + 0.995 \cdot G_{\rm LI}$.

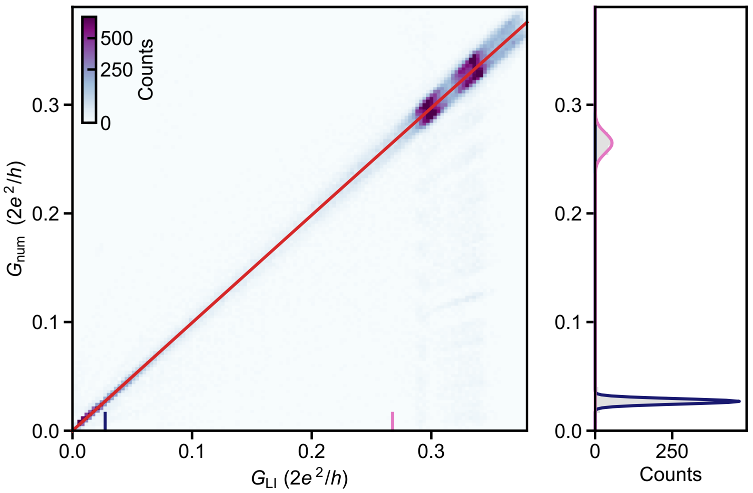

Supplementary Figure 20. Calibration function extracted from the conductance data of the N–S junction presented in Fig. 4 of the main text. Numerical differential conductance, $G_{\rm num}$, vs. AC differential conductance, $G_{\rm LI}$. Here, the lock-in frequency is $f = 23\,\text{Hz}$.

Supplementary Figure 21 shows the calibration for the left junction of the correlation device in Fig. 5e of the main text. In Supplementary Figure 22, the calibration is presented for the right junction of the correlation device in Fig. 5f of the main text. This is the only N–S junction device that was measured at a relatively large lock-in frequency ($f = 72\,\text{Hz}$). The right panels of Supplementary Figure 21 and Supplementary Figures 22a–c show exemplary fits of the histograms using a Gaussian. The red traces represent the fitting by the least-squares method using the polynomial regression function $G_{\rm num} = A \cdot G_{\rm LI}^2 + B \cdot G_{\rm LI} + C$. The mapping in Supplementary Figure 21 yields the parametrization $G_{\rm num} = -0.108 \cdot G_{\rm LI}^2 + 1.043 \cdot G_{\rm LI} - 0.003$. In Supplementary Figure 22, the weighted average of the fitting functions yields the mapping function $G_{\rm num} = 0.023 \cdot G_{\rm LI}^2 + 1.034 \cdot G_{\rm LI} - 0.040$, where the residuals of the individual measurements provide the weights. Supplementary Figure 22d summarizes the parabolic (A) and linear (B) fit parameters from Supplementary Figures 22a–c. The black data point indicates the weighted average of the fit parameters.

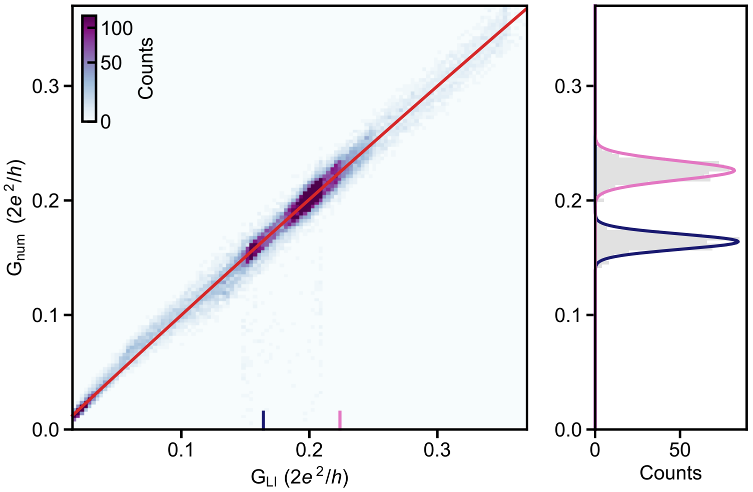

Supplementary Figure 21. Calibration function extracted from the conductance data of the N–S junction presented in Fig. 5e of the main text. Numerical differential conductance, G_{num}, vs. AC differential conductance, G_{LI}. Here, the lock-in frequency is $f = 23\,\mathrm{Hz}$.

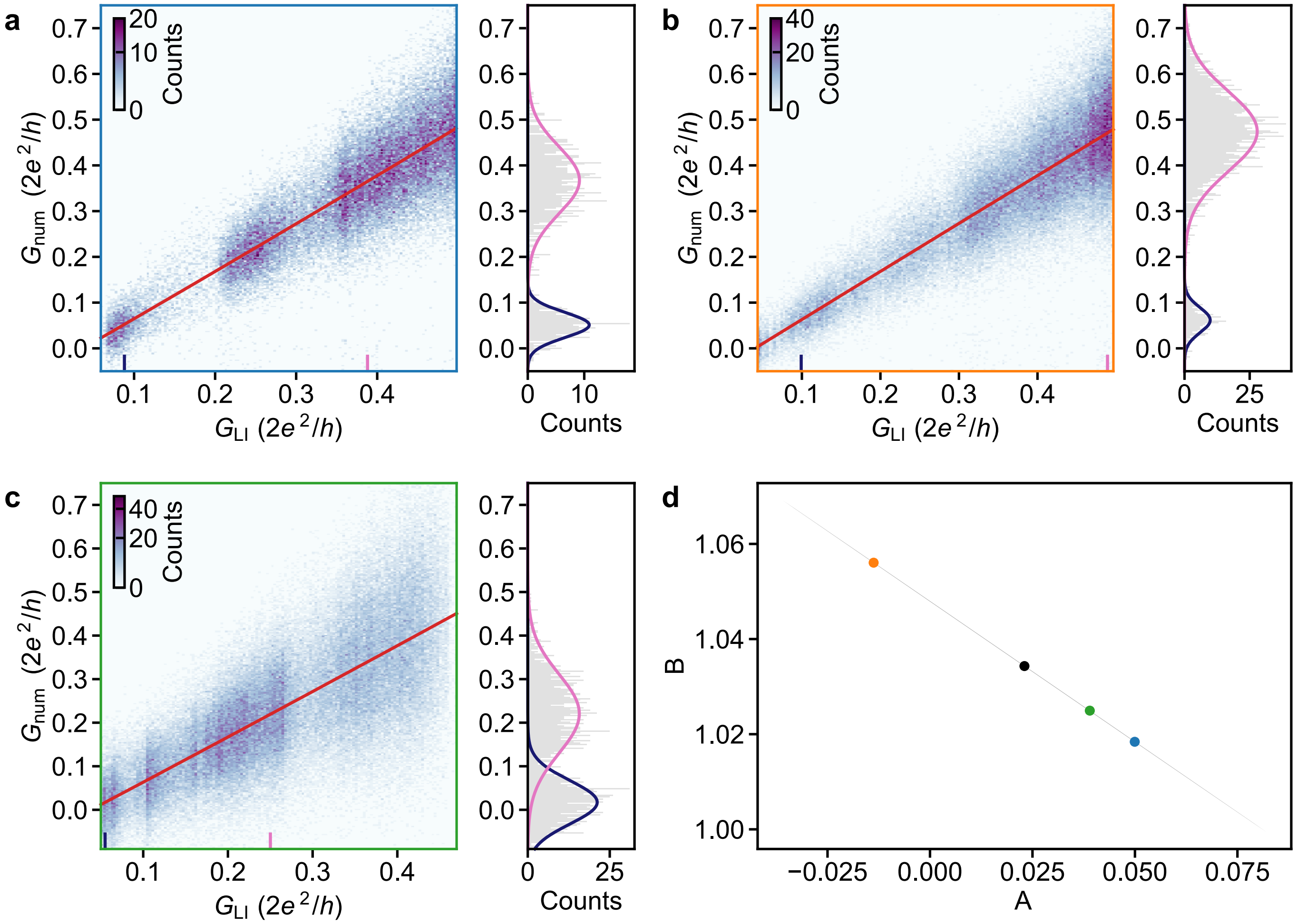

Supplementary Figure 22. Calibration functions extracted for the N–S junction presented in Fig. 5f of the main text. **a**–**c** Numerical differential conductance, G_{num}, vs. AC differential conductance, G_{LI}. Each of the three panels is from separate data set. Here, the lock-in frequency is $f = 72\,\mathrm{Hz}$. **d** Summary of the parabolic (A) and linear (B) fit parameters in panels (**a**–**c**). The colors of the data points correspond to the axis colors of the respective panels. The black data point denotes the weighted average fit parameters, where the weights are determined by the residuals of the individual fits. The grey area designates the 95% confidence interval.

List of N–S Devices

The specifications of the N–S junction devices studied in this work are listed in Supplementary Table 3. All devices are fabricated by evaporating an Al thin film at 30° with respect to the substrate plane.

**N–S device**	**Sample ID/ device name**	**Evaporation angle**	**Nanowire diameter (nm)**	**Maximum Al thickness (nm)**	**Oxidation**
device 1	U53/1.3	30°	100	16	in O_2 atmosphere
device 2	U53/1.1	30°	100	16	in O_2 atmosphere
device 3	U48/2.3	30°	105	16	in O_2 atmosphere
device 4	U53/1.2, left	30°	80	16	in O_2 atmosphere
device 5	U53/1.2, right				

Supplementary Table 3. Summary of the N–S devices presented in this study. Device 1 is the sample presented in Fig. 4 of the main text. Devices 4 and 5 correspond to the left and right N–S junctions, respectively, of the sample presented in Fig. 5 of the main text.

N–S Junction Spectroscopy

Deep in the tunnelling regime, the subgap conductance is strongly suppressed. As illustrated in Supplementary Figure 23, the ratio of the above-gap conductance and the subgap conductance is approximately a factor of 100. In Supplementary Figures 23a,b, the differential conductance line-cut for N–S device 1 (i.e. the same device as in Fig. 4 of the main text) is fitted using the BCS–Dynes term (red) and the BTK model (green). The data in Supplementary Figures 23c,d shows a line-cut for another N–S junction (device 2), which is not presented in the main text. The fitting parameters of the BTK model are the induced gap, Δ_{ind}, the normal-state conductance, G_{N}, and the temperature, T. For device 1 it yields an induced gap of $\Delta_{\mathrm{ind}} = 231\,\mu\mathrm{eV}$ and for device 2 the extracted gap is $\Delta_{\mathrm{ind}} = 241\,\mu\mathrm{eV}$. In the BTK model the only effective broadening parameter is the temperature, which for both devices yields $T \approx 0.1\,\mathrm{K}$. The two N–S junctions presented in Fig. 5 of the main text (devices 4 and 5) also exhibit comparable values of the induced gap of $\Delta_{\mathrm{ind}} \sim 230$–$240\mu\mathrm{eV}$.

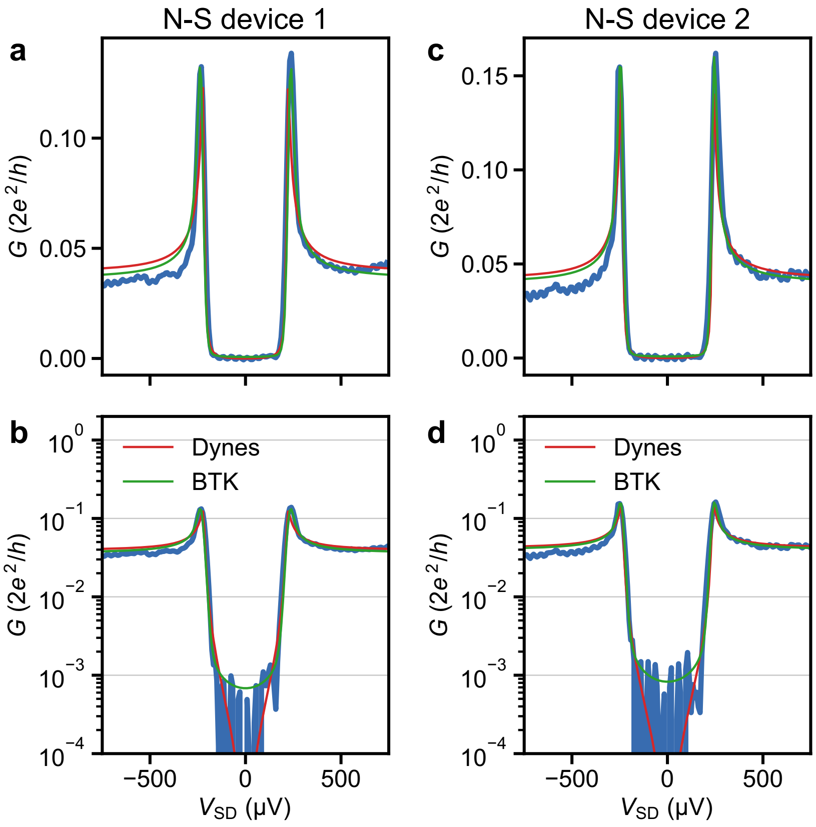

Supplementary Figure 23. **a**, **b** Differential conductance vs. source–drain voltage line-cut for N–S device 1 (same as in Fig. 4 of the main text) on a linear scale in (**a**) and on a logarithmic scale in (**b**). Here, the tunnel-gate voltage is $V_{\mathrm{TG}} = 0.530\,\mathrm{V}$ and the super-gate voltage is $V_{\mathrm{SG}} = 0\,\mathrm{V}$. **c**, **d** Differential conductance vs. source–drain voltage line-cut for N–S device 2 (not presented in the main text) on a linear scale in (**c**) and on a logarithmic scale in (**d**). Here, the tunnel-gate voltage is $V_{\mathrm{TG}} = 2.004\,\mathrm{V}$ and the super-gate voltage is $V_{\mathrm{SG}} = 7.0\,\mathrm{V}$. The fit of the BCS–Dynes term and of the BTK model are shown in red and green, respectively.

Temperature Dependence of the Induced Gap

In Supplementary Figure 24, we present the temperature dependence for another device (N–S device 3), which is not presented in the main text. In the limit $k_\mathrm{B}T \ll \Delta_\mathrm{ind}$, the subgap conductance, G_S, scales with temperature, T, as [17]

$$G_\mathrm{S}\left(V_\mathrm{SD}=0\right) = G_\mathrm{N}\sqrt{\frac{2\pi\Delta_\mathrm{ind}}{k_\mathrm{B}T}}e^{-\Delta_\mathrm{ind}/k_\mathrm{B}T}, \tag{12}$$

where G_N is the normal-state conductance and k_B is the Boltzmann constant. The purple trace in Supplementary Figure 24a measured at $T = 18\,\mathrm{mK}$ is well described by the BTK model with an induced gap of $\Delta_\mathrm{ind} = 237\,\mu\mathrm{eV}$. This is very similar to the magnitude of the induced gap of the other two N–S devices shown in Supplementary Figure 23, albeit those junctions are formed in a separate Al deposition step on another substrate. The theoretical model in equation (12) can describe the smearing of the density of states with temperature. It yields a fit parameter of $\Delta_\mathrm{ind} \approx 210\,\mu\mathrm{eV}$, which is a bit smaller than the gap directly extracted from the tunnelling spectroscopy.

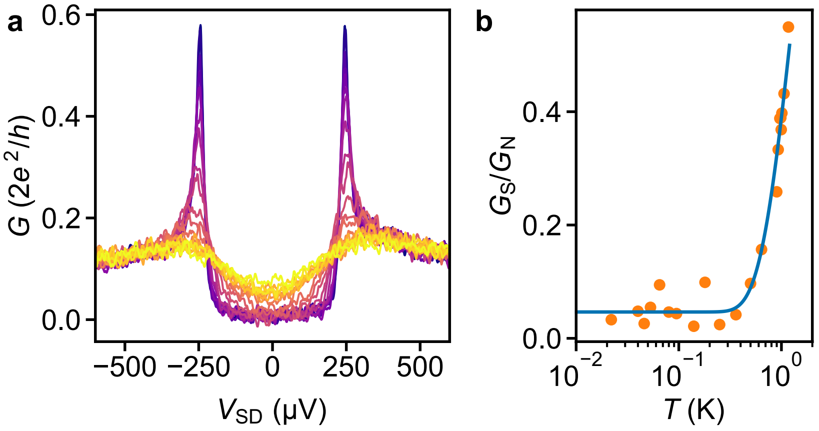

Supplementary Figure 24. Temperature dependence of the induced gap (N–S device 3). **a** Tunnelling conductance vs. source–drain voltage between $T = 18\,\mathrm{mK}$ (purple) and $T = 1.17\,\mathrm{K}$ (yellow). **b** Subgap conductance averaged between $V_\mathrm{SD} = \pm 30\mu\mathrm{V}$ (G_S) divided by the normal-state conductance (G_N) as a function of T. The blue trace is a fit to equation (12).

Hard Induced Gap

In Supplementary Figure 25, we report the fit of the BTK model to the data shown in Fig. 4b of the main text (N–S device 1). The extracted induced superconducting gap is $\Delta_{\mathrm{ind}} \sim 230\,\mu\mathrm{eV}$.

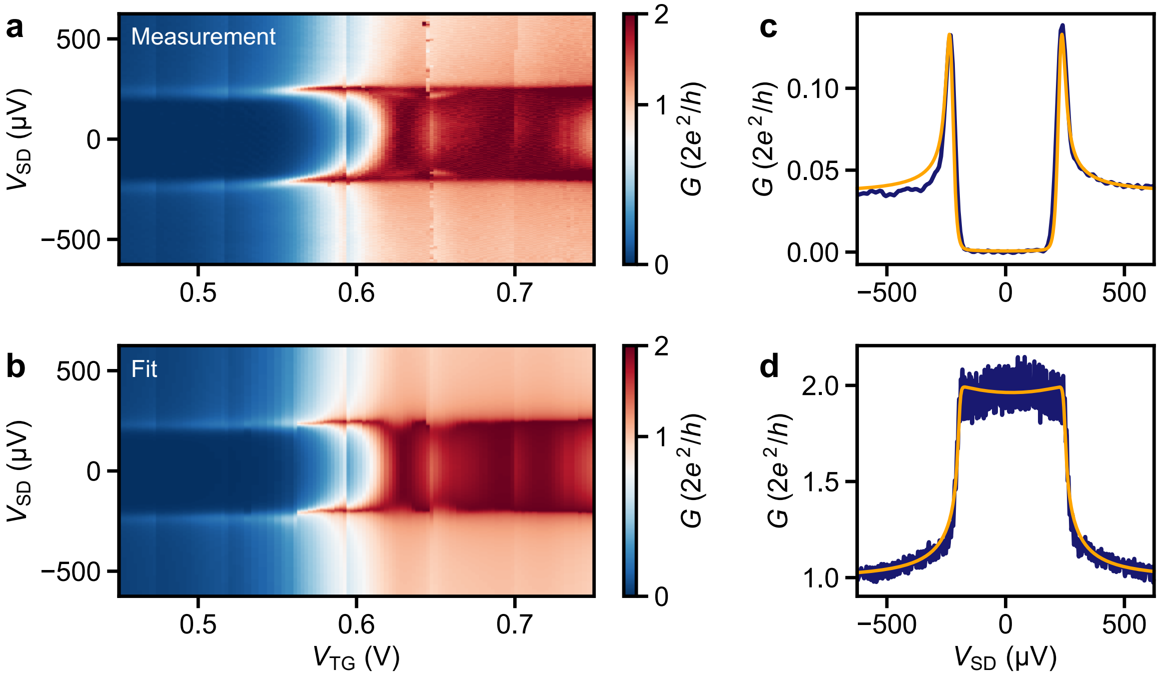

Supplementary Figure 25. N–S junction voltage-bias spectroscopy and the corresponding fit of the BTK model [18] for N–S device 1. **a** Differential conductance, G, as a function of source–drain voltage, V_{SD}, and bottom tunnel-gate voltage, V_{TG}, from Fig. 4b of the main text. **b** Fit of the BTK model to the data set in panel (**a**). The fit parameters include the induced gap, the temperature, and the barrier strength Z, wich is given by the transmission $(1 + Z^2)^{-1}$. **c**, **d** Line-cut of the data in panel (**a**) (dark blue) at $V_{\mathrm{TG}} = 0.53\,\mathrm{V}$ and at $V_{\mathrm{TG}} = 0.69\,\mathrm{V}$, respectively. The orange traces show the corresponding fits to the BTK model.

Zero-Bias Peaks in the N–S Device

In Supplementary Figure 26, we present additional data for the first N–S device (cf. Fig. 4 of the main text) in a parallel magnetic field for two different super-gate voltages. In the main text, we present ballistic transport and pronounced Andreev enhancement for the same N–S device. Here, we show the evolution of discrete subgap states as a function of magnetic field and the formation of ZBPs, which in some cases can reach a conductance close to $2e^2/h$.

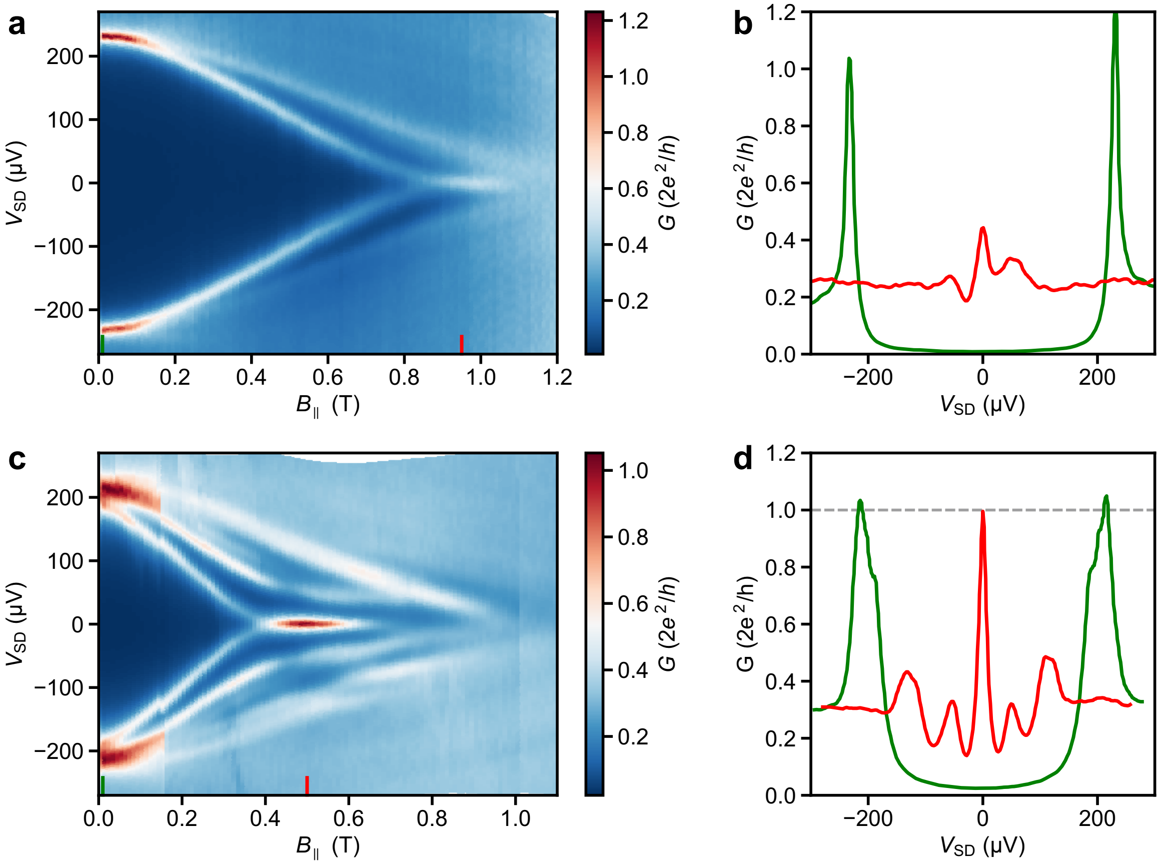

Supplementary Figure 26. Magnetic-field-dependent voltage-bias spectroscopy for N–S device 1 from Fig. 4 of the main text, demonstrating the formation of zero-bias peaks in the differential conductance. **a** G as a function of V_{SD} and $B_\parallel$. The super-gate voltage is $V_{\mathrm{SG}} = 7.5\,\mathrm{V}$ and the tunnel-gate voltage is $V_{\mathrm{TG}} = 0.5\,\mathrm{V}$. **b** Line-cuts from panel (**a**) at the positions indicated by the two lines. **c** G as a function of V_{SD} and $B_\parallel$. Here, $V_{\mathrm{SG}} = 2.97\,\mathrm{V}$ and $V_{\mathrm{TG}} = 0.417\,\mathrm{V}$. **d** Line-cuts from panel (**c**) at the positions indicated by the two lines.

Zero-Bias Peaks and Super-Gate Dependence

Additional N–S spectroscopy measurements of the left N–S junction of the device presented in Fig. 5 of the main text are shown in Supplementary Figure 27. Here, the voltage at the super gate – the bottom gate controlling the electrochemical potential in the hybrid nanowire segment – is larger ($V_{SG} = 0.525\,V$ vs. $0\,V$). The differential conductance vs. V_{SD} and $B_{\|}$ is depicted in Supplementary Figure 27a, the bias-voltage line-cut in Supplementary Figure 27b illustrates the pronounced zero-bias conductance peak at large magnetic fields. However, the magnitude of the ZBP conductance depends on the tuning of the tunnel-gate and super-gate voltages (cf. Supplementary Figure 27c).

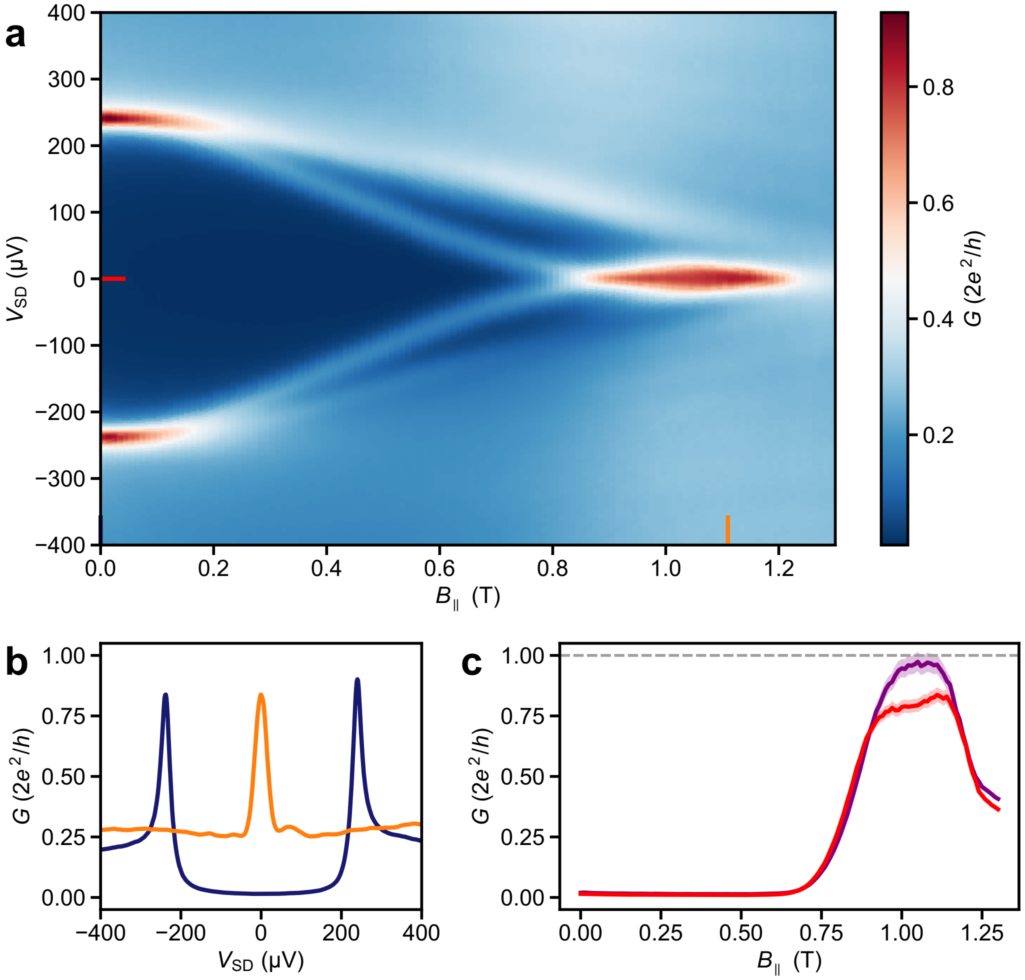

Supplementary Figure 27. Voltage-bias spectroscopy of a subgap state with a large zero-bias peak conductance close to $2e^2/h$ (measured at the left N–S junction of the device presented in Fig. 5 of the main text). Here, the super-gate voltage $V_{SG} = 0.525\,V$. **a** Differential conductance, G, as a function of the bias voltage at the left terminal, V_{SD}, and the magnetic field along the wire axis. **b** Voltage-bias line-cut of the differential conductance at zero field (blue) and at $B_{\|} = 1.11\,T$ (orange). **c** G vs. $B_{\|}$ line-cuts at $V_{SD} = 0\,\mu V$ from panel (**a**) (red, at $V_{SG} = 0.525\,V$) and from Fig. 5e of the main text (purple, at $V_{SG} = 0\,V$). The shaded areas behind the solid traces correspond to the variation in conductance assuming an uncertainty of $\pm 0.5\,k\Omega$ in estimating the actual series resistance.

In Supplementary Figure 28 additional data from the high-field regime are presented (here $B_{\parallel} = 0.85 - 1.15\,\mathrm{T}$). For the same bottom-gate settings as in Supplementary Figure 27 we observe ZBPs that emerge concurrently at both boundaries of the superconductor–semiconductor nanowire segment (cf. Supplementary Figures 28a,b). By fixing the magnetic field at $B_{\parallel} = 1.0\,\mathrm{T}$ we can observe the evolution of the ZBPs at the left and right N–S junctions as a function of the voltage on the super gate underneath the hybrid nanowire segment (see Supplementary Figures 28c,d). The asymmetry in the conductance of Supplementary Figure 28d with respect to bias polarity is presumably related to energy-dependent tunnel barrier transmission at the right N–S junction.
The concurrent evolution of the ZBPs at both N–S boundaries of the correlation device as a function of the super-gate voltage is also depicted in Supplementary Figure 29 for same tunnel-gate settings as in Fig. 5 of the main text.

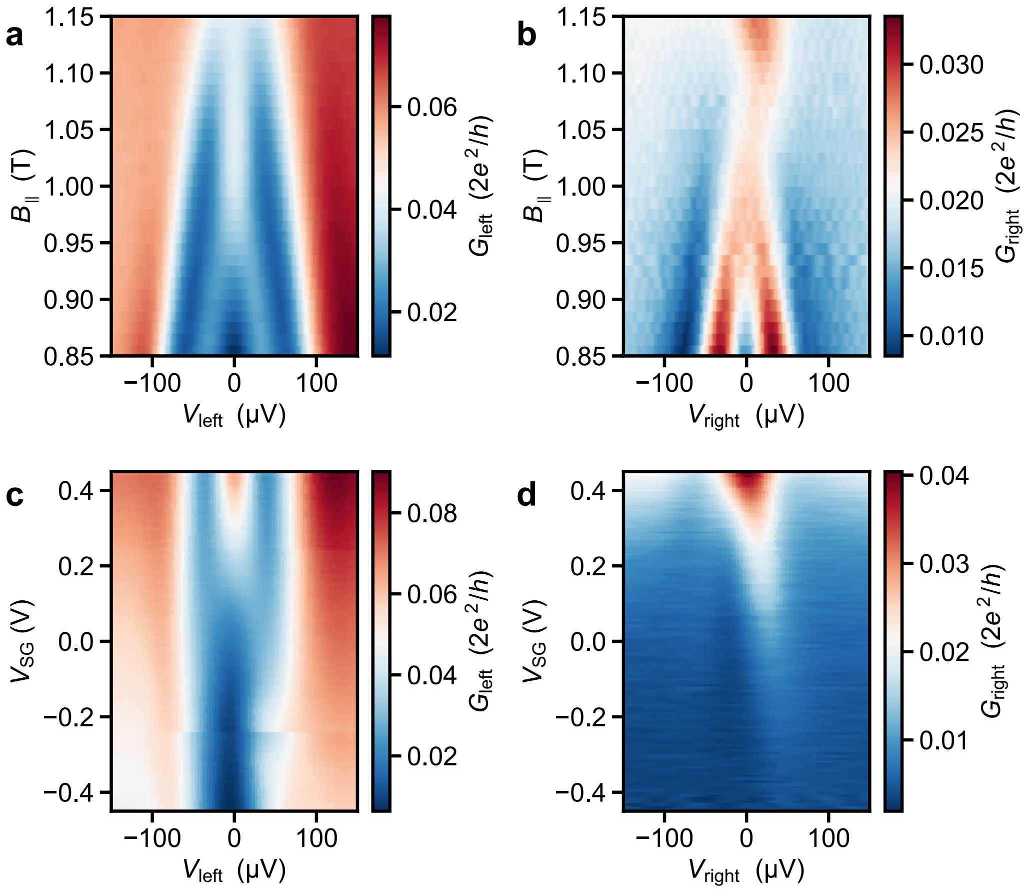

Supplementary Figure 28. Simultaneous appearance of zero-bias peaks at both hybrid boundaries (same device as in Fig. 5 of the main text). The two tunnel gates are set to $V_{\mathrm{TG,left}} = 0.47\,\mathrm{V}$ and $V_{\mathrm{TG,right}} = 0.13\,\mathrm{V}$. **a**, **b** Differential conductance, $G_{\mathrm{left/right}}$, as a function of magnetic field, $B_{\parallel}$, and bias voltage at the left and right terminal, respectively. Here, the super-gate voltage $V_{\mathrm{SG}} = 0.525\,\mathrm{V}$, i.e. identical as for the data in Supplementary Figure 27a. **c**, **d** Differential conductance, $G_{\mathrm{left/right}}$, at $B_{\parallel} = 1.0\,\mathrm{T}$ as a function of V_{SG} and bias voltage at the left and right terminal, respectively.

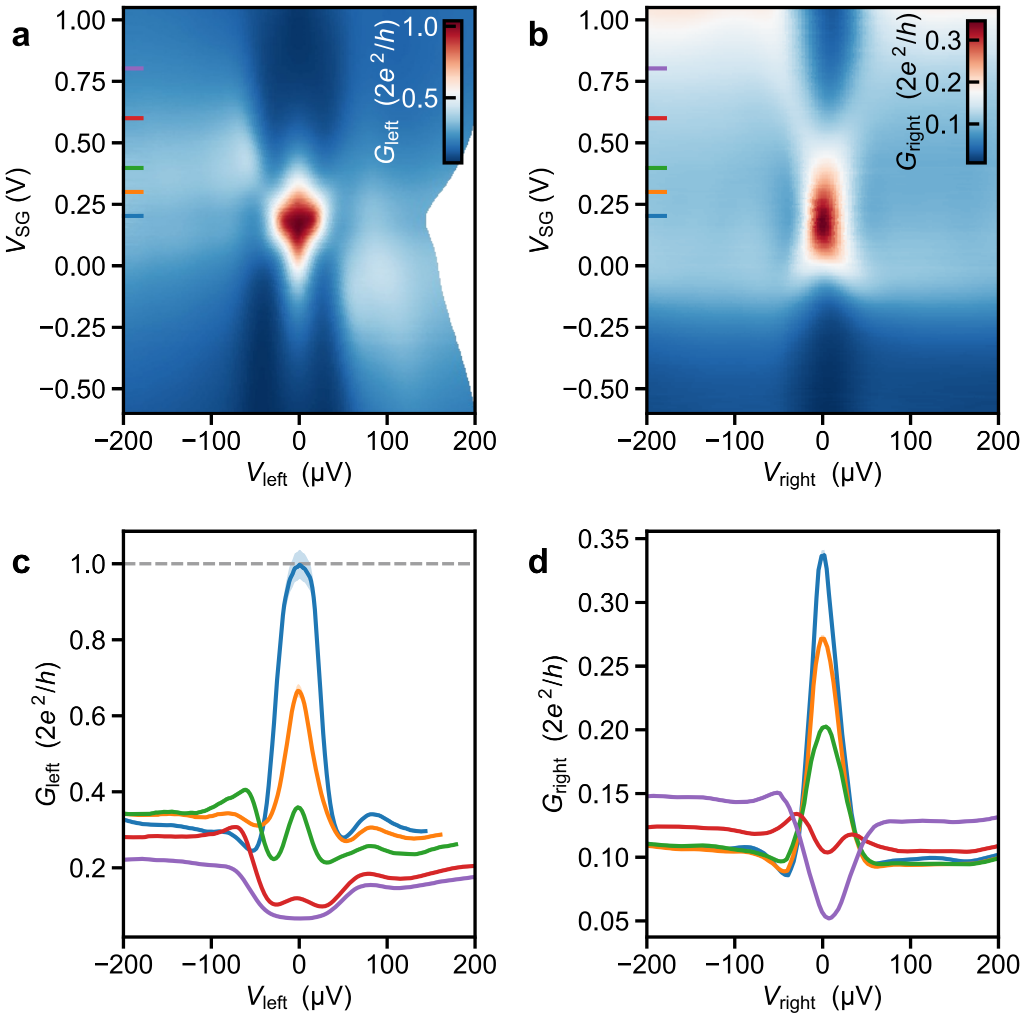

Supplementary Figure 29. Simultaneous appearance of zero-bias peaks at both hybrid boundaries (same device and same tunnel-gate settings as in Fig. 5 of the main text). The two tunnel gates are set to $V_{\mathrm{TG,left}} = 0.52\,\mathrm{V}$ and $V_{\mathrm{TG,right}} = 0.21\,\mathrm{V}$. **a**, **b** Differential conductance, $G_{\mathrm{left/right}}$, at $B_{\parallel} = 1.0\,\mathrm{T}$ as a function of V_{SG} and bias voltage at the left and right terminal, respectively. **c**, **d** Line-cuts from panels (**a**) and (**b**) at the values of V_{SG} designated by the coloured lines. The shaded areas behind the solid traces correspond to the variation in conductance assuming an uncertainty of $\pm 0.5\,\mathrm{k\Omega}$ in estimating the actual series resistance.

SUPPLEMENTARY NOTE 5. REALIZATION OF ADVANCED HYBRID DEVICES

In this section, we present another example of more advanced nanowire devices that can be realized using the shadow-wall technique. In the main text, we have introduced the necessary ingredients to realize the basic implementation of a topological qubit using the shadow-wall technique. In Supplementary Figure 30, we show another application of the shadow-wall concept, which is intended as an experimental implementation of a theoretical proposal by Schrade and Fu [19]. It represents a superconducting quantum interference device (SQUID) formed by two InSb nanowires (green) placed deterministically in close vicinity of shadow walls (blue). Previous realizations of nanowire SQUIDs relied on electron-beam lithography and standard lift-off technique [20]. Here, top gates (yellow) are fabricated to form a single Josephson junction (JJ) on the left side of the device and a superconducting island is defined by two tunnel gates and one plunger gate on the right side of the device. Source and drain electrodes are created by bonding directly to the Al film (grey) at the bottom and at the top of the SQUID loop, respectively. By utilizing shadow-wall substrates with bottom gates, this SQUID sample can be realized without any post-interface fabrication steps.

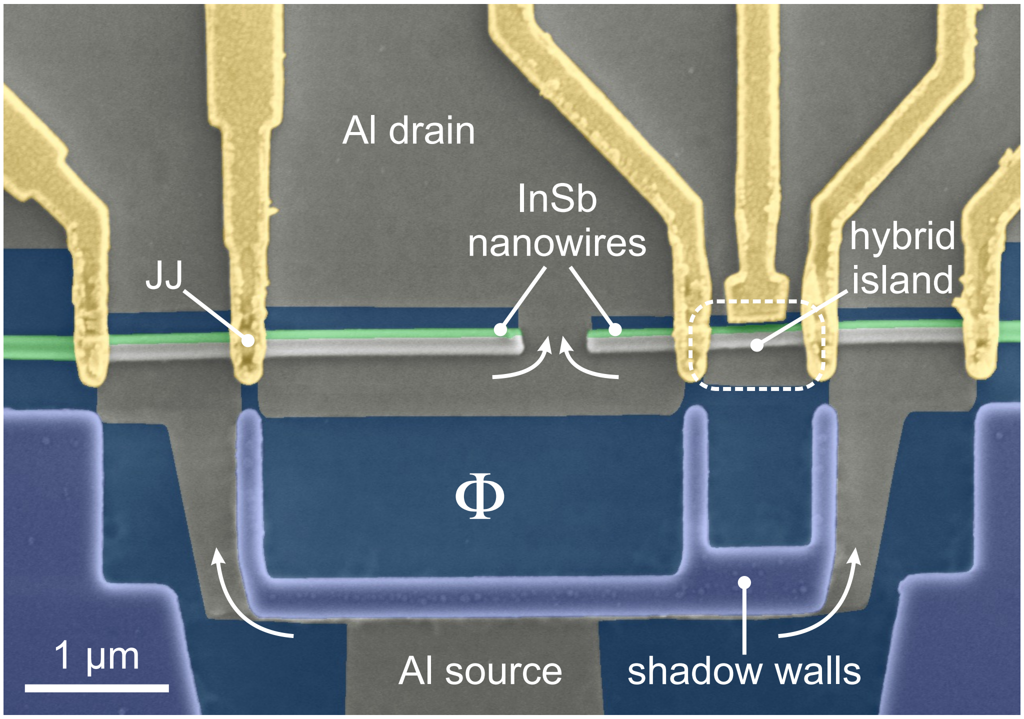

Supplementary Figure 30. SQUID sample formed by placing two InSb nanowires next to each other in the shadow region of the dielectric walls. Electrical current flows from source to drain via the Josephson junction (denoted as JJ) and the hybrid charge island as indicated by the white arrows. The magnetic flux threading through the SQUID loop is denoted as Φ. The bottom of the SQUID loop is partly formed by the Al thin film covering the side of the central shadow wall.

SUPPLEMENTARY REFERENCES

[1] K. Flöhr, M. Liebmann, K. Sladek, H. Y. Günel, R. Frielinghaus, F. Haas, C. Meyer, H. Hardtdegen, Th. Schäpers, D. Grützmacher, and M. Morgenstern. Manipulating InAs nanowires with submicrometer precision. *Rev. Sci. Instrum.*, 82(11):113705, 2011.

[2] J. L. Webb, J. Knutsson, M. Hjort, S. Gorji Ghalamestani, K. A. Dick, R. Timm, and A. Mikkelsen. Electrical and surface properties of InAs/InSb nanowires cleaned by atomic hydrogen. *Nano Lett.*, 15(8):4865–4875, 2015.

[3] L. Haworth, J. Lu, D. I. Westwood, and J. E. MacDonald. Atomic hydrogen cleaning, nitriding and annealing InSb (100). *Appl. Surf. Sci.*, 166(1):253 – 258, 2000.

[4] R. Tessler, C. Saguy, O. Klin, S. Greenberg, E. Weiss, R. Akhvlediani, R. Edrei, and A. Hoffman. Oxide-free InSb (100) surfaces by molecular hydrogen cleaning. *Appl. Phys. Lett.*, 88:1918–031918, Jan 2006.

[5] L. Dong, R. W. Smith, and D. J. Srolovitz. A two-dimensional molecular dynamics simulation of thin film growth by oblique deposition. *J. Appl. Phys.*, 80:5682 – 5690, 1997.

[6] A. Barranco, A. Borras, A. R. Gonzalez-Elipe, and A. Palmero. Perspectives on oblique angle deposition of thin films: From fundamentals to devices. *Prog. Mater. Sci.*, 76:59 – 153, 2016.

[7] A. E. Antipov, A. Bargerbos, G. W. Winkler, B. Bauer, E. Rossi, and R. M. Lutchyn. Effects of gate-induced electric fields on semiconductor Majorana nanowires. *Phys. Rev. X*, 8:031041, Aug 2018.

[8] F. K. de Vries, M. L. Sol, S. Gazibegovic, R. L. M. op het Veld, S. C. Balk, D. Car, E. P. A. M. Bakkers, L. P. Kouwenhoven, and J. Shen. Crossed Andreev reflection in InSb flake Josephson junctions. *Phys. Rev. Res.*, 1:032031, Dec 2019.

[9] S. Gazibegovic, G. Badawy, T. L. J. Buckers, P. Leubner, J. Shen, F. K. de Vries, S. Koelling, L. P. Kouwenhoven, M. A. Verheijen, and E. P. A. M. Bakkers. Bottom-up grown 2D InSb nanostructures. *Adv. Mater.*, 31(14):1808181, 2019.

[10] M. W. A. de Moor, J. D. S. Bommer, D. Xu, G. W. Winkler, A. E. Antipov, A. Bargerbos, G. Wang, N. van Loo, R. L. M. Op het Veld, S. Gazibegovic, D. Car, J. A. Logan, M. Pendharkar, J. S. Lee, E. P. A. M. Bakkers, C. J. Palmstrøm, R. M. Lutchyn, L. P. Kouwenhoven, and H. Zhang. Electric field tunable superconductor-semiconductor coupling in Majorana nanowires. *New J. Phys.*, 20(10):103049, Oct 2018.

[11] D. Averin and A. Bardas. ac Josephson effect in a single quantum channel. *Phys. Rev. Lett.*, 75(9):1831–1834, Aug 1995.

[12] A. Bardas and D. V. Averin. Electron transport in mesoscopic disordered superconductor–normal-metal–superconductor junctions. *Phys. Rev. B*, 56(14):R8518–R8521, Oct 1997.

[13] M. P. Nowak, M. Wimmer, and A. R. Akhmerov. Supercurrent carried by nonequilibrium quasiparticles in a multiterminal Josephson junction. *Phys. Rev. B*, 99(7):075416, Feb 2019.

[14] B. Nijholt, J. Weston, J. Hoofwijk, and A. Akhmerov. *Adaptive*: parallel active learning of mathematical functions, 10.5281/zenodo.3475095, 2019.

[15] D. E. Morris and M. Tinkham. Effect of magnetic field on thermal conductivity and energy gap of superconducting films. *Phys. Rev. Lett.*, 6:600–602, Jun 1961.

[16] V. S. Mathur, N. Panchapakesan, and R. P. Saxena. Magnetic-field dependence of the energy gap in superconductors. *Phys. Rev. Lett.*, 9:374–375, Nov 1962.

[17] M. Tinkham. *Introduction to superconductivity.* Dover Publications, 1996.

[18] G. E. Blonder, M. Tinkham, and T. M. Klapwijk. Transition from metallic to tunneling regimes in superconducting microconstrictions: Excess current, charge imbalance, and supercurrent conversion. *Phys. Rev. B*, 25:4515–4532, Apr 1982.

[19] C. Schrade and L. Fu. Andreev or Majorana, Cooper finds out. *arXiv e-prints arXiv:1809.06370*, Sep 2018.

[20] D. B. Szombati, S. Nadj-Perge, D. Car, S. R. Plissard, E. P. A. M. Bakkers, and L. P. Kouwenhoven. Josephson φ_0-junction in nanowire quantum dots. *Nat. Phys.*, 12:568–572, 2016.
